# Immunological Basis of the Endometriosis: The Complement System as a Potential Therapeutic Target

**DOI:** 10.3389/fimmu.2020.599117

**Published:** 2021-01-11

**Authors:** Chiara Agostinis, Andrea Balduit, Alessandro Mangogna, Gabriella Zito, Federico Romano, Giuseppe Ricci, Uday Kishore, Roberta Bulla

**Affiliations:** ^1^Institute for Maternal and Child Health, IRCCS (Istituto di Ricovero e Cura a Carattere Scientifico) “Burlo Garofolo”, Trieste, Italy; ^2^Department of Life Sciences, University of Trieste, Trieste, Italy; ^3^Department of Medical, Surgical and Health Science, University of Trieste, Trieste, Italy; ^4^Biosciences, College of Health, Medicine and Life Sciences, Brunel University London, Uxbridge, United Kingdom

**Keywords:** endometriosis, inflammation, innate immunity, complement system, immunotherapy

## Abstract

Endometriosis (EM) is a chronic disease characterized by the presence and proliferation of functional endometrial glands and stroma outside the uterine cavity. Ovaries and pelvic peritoneum are the most common locations for endometrial ectopic tissue, followed by deep infiltrating EM sites. The cyclic and recurrent bleeding, the progressive fibrosis and the peritoneal adhesions of ectopic endometrial glands, may cause different symptoms depending on the origin involved. EM is a frequent clinical condition affecting around 10% of women of mainly reproductive age, as well as in post-menopausal women and adolescents, especially with uterine anomalies. The risk of developing EM depends on a complex interaction between genetic, immunological, hormonal, and environmental factors. It is largely considered to arise due to a dysfunction of immunological surveillance. In fact, women with EM exhibit altered functions of peritoneal macrophages, lymphocytes and natural killer cells, as well as levels of inflammatory mediators and growth factors in the peritoneal fluid. In EM patients, peritoneal macrophages are preponderant and highly active compared to healthy women. Peritoneal macrophages are able to regulate the events that determine the production of cytokines, prostaglandins, growth factors and complement components. Several studies have shown alteration in the regulation of the complement activation, leading to chronic inflammation characteristic of EM. Aberrant regulation/activation of the complement system has been observed in the peritoneal cavity of women affected by EM. Thus, complement inhibition may represent a new approach for the treatment of EM, given that a number of complement inhibitors are under pre-clinical and clinical development. Such an intervention may provide a broader therapeutic control of complement-mediated inflammatory damage in EM patients. This review will focus on our current understanding of the role of complement activation in EM and possible modalities available for complement-based therapy.

## Introduction

Endometriosis (EM) is a common inflammatory disease caused by the dissemination or growth of endometrium-like tissue at abnormal locations, or through the onset of endometrial tissue *via* metaplasia outside the usual location (1, 2). The disease is considered a heterotopia; the endometrium-like heterotopic tissues are characterized by glands and stroma functionally responsive to local, endogenous and exogenous hormonal stimuli (3). In fact, the ectopic endometrium is affected, like the normal uterine mucosa, by the ovarian hormones, especially estrogens, and therefore, become proliferative and functional (characterized by flaking and bleeding during the menstrual period) similar to those that occur in the normal endometrium (3). It is, therefore, a disease invariably of fertile age when ovarian activity is present; it occurs exceptionally in puberty and rarely in adolescence. It tends to regress in post-menopause or after castration. It is more frequent in nulliparous women (4, 5).

**Figure 1:**
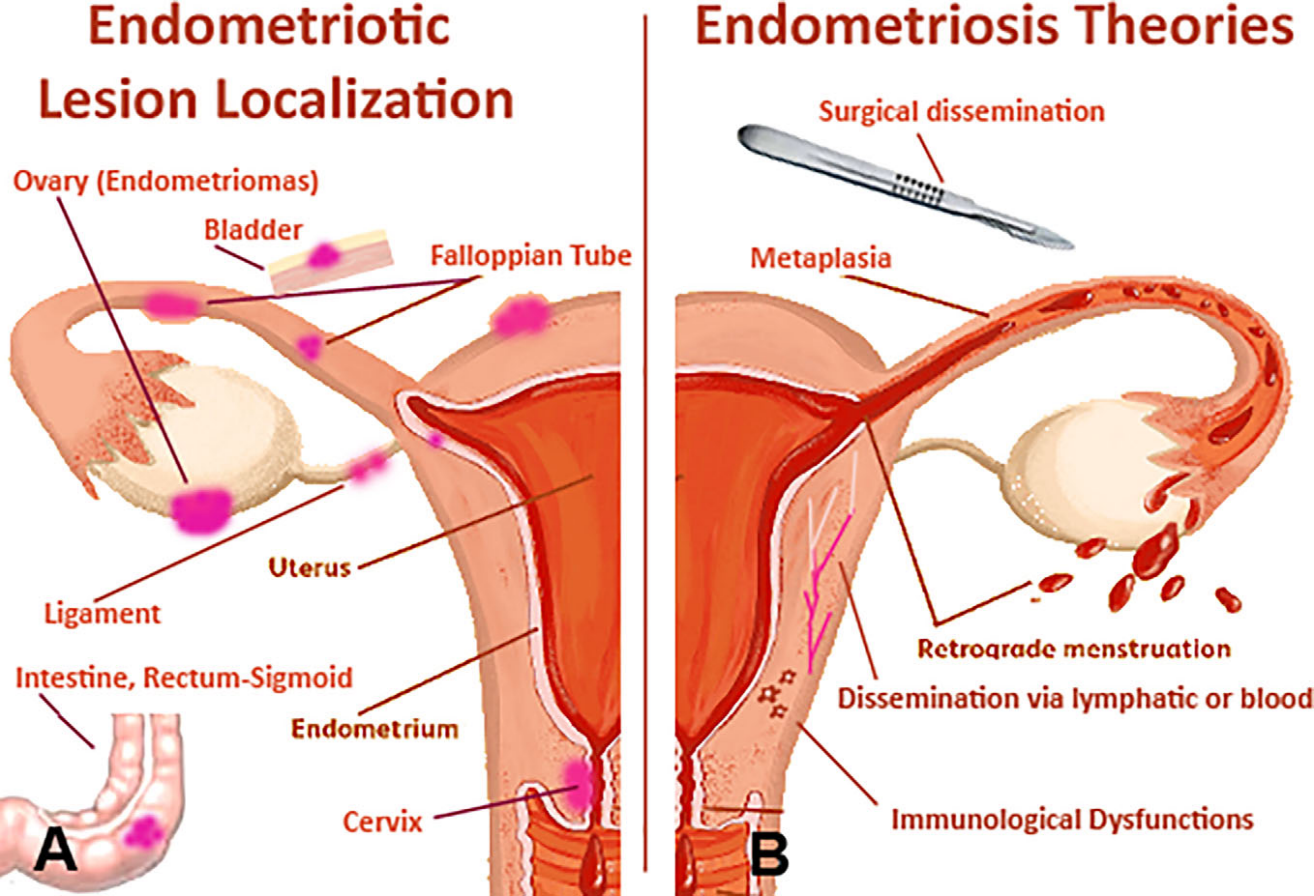
Pathophysiological features of endometriosis (EM). **(A)** the female anatomy with areas that are commonly affected by EM. **(B)** likely pathogenic mechanisms involved in EM: potential origins of the endometriotic lesions include surgical dissemination, transplantation of endometrial tissue through retrograde menstruation, and *in situ* coelomic metaplasia of the peritoneal lining. Vascular or lymphatic metastasis occurs rarely, in cases of extrapelvic lesions. Superficial and deep endometriotic lesions are established and maintained through interacting molecular mechanisms that promote cellular adhesion and proliferation, systemic and localized steroidogenesis, localized inflammatory response and immune dysregulation.

The ectopic EM is usually found on the pelvic peritoneum and in the pelvic organs (ovaries, tubes, intestine, rectum-sigmoid, uterine ligaments, recto-vaginal septum, and bladder) (6). EM can also occur in organs and tissues outside or far from the pelvis (navel, vulva, scars of laparotomy operations, appendix, and lungs) (6, 7). The etiology of EM remains unclear (8). Even though the precise frequency of EM in the general population is unknown, it represents a recurrent pathology among women of reproductive age (2). The estimates of the incidence of the disease (which can vary enormously) are around 10% in reproductive-age women (4). Endometriotic lesions, in particular ovary endometrioma (**Figure 1**), present a 2–3-fold increased risk of transformation in clear-cell or endometrioid ovarian carcinomas. Recent findings have demonstrated that somatic mutations in cancer-associated genes, such as *KRAS, PIK3CA, ARID1A*, and *PPP2R1A*, are commonly found in different types of EM (9). EM is an estrogen-dependent disease since estrogen appears to play a primary role in the development and maintenance of endometriotic lesions (10). Several proposals have been put forward to explain the pathogenic mechanisms involved in EM (2) (**Figure 1**), which are as follows:

### Tubal Reflux of Menstrual Blood and Implantation of Endometrial Frustules in Various Tissues

Frustules of uterine mucosa not only have the ability to implant, but can be stimulated to proliferate by the cyclic action of ovarian estrogens. Since retrograde menstruation is so frequent (almost considered a physiological phenomenon), the possibility of developing EM is likely to depend on the relationship between the quantity of endometrium refluxed into the peritoneal cavity, and the receptivity intrinsic to the implant of endometrial cells (11, 12).

### Dissemination *via* Lymphatic or Blood

Viable endometrial cells could enter the blood vessels and lymphatics with consequent embolization and implantation at ectopic sites (13–15).

### Metaplasia of the Epithelium of Celomatic or Müllerian Origin

Ectopic EM can originate from mesothelial totipotent cells of the peritoneum through metaplasia. This notion is considerably sound due to the fact that the celomatic epithelium, from which the epithelial cells of the Müllerian ducts originate, also differs in pleural and peritoneal epithelial cells, as well as in cells of the ovarian surface. Novak was the first to suggest the possibility that metaplasia could be prompted by induction factors (11, 16), such as sexual hormones, tubal reflux of endometrial debris, and inflammatory processes.

### Surgical Dissemination

Surgical dissemination seems likely to be responsible for endometrial tissue spreading to ectopic sites, for instance on laparotomy scars. The so-called “iatrogenic endometriosis” may occur after operations of the uterine cavity (myomectomy and metroplasty), or in the case of surgical interventions carried out on pelvic organs, which may account for localization at the vulvo-perineal level (17, 18).

### Dysfunctional Immune System

Studies have also suggested that EM is due to an alteration in the immune system in terms of immune-cell recruitment, cell-adhesion, and upregulated inflammatory processes, which can facilitate the implantation and survival of endometriotic lesions (19, 20). A distinct epigenetic profile can be observed between eutopic and ectopic endometrial tissues. By analysing global promoter methylation patterns, researchers have demonstrated that differentially methylated genes are associated with immune surveillance, inflammatory response, cell adhesion and negative regulation of apoptosis (21).

There is a paramount role of immune cells in the pathogenesis of EM. Recently, it has been shown that the complement system is one of the most preponderant pathways impaired in the EM (20, 22, 23). In this review, we examine how immune cells and complement contribute to the development and maintenance of EM, and possible modalities available for complement-based therapy in the clinical practice.

## Endometriotic Immune Microenvironment

### Monocytes and Macrophages

Macrophages are in the protagonist immune cells in the pathogenesis of EM. In normal endometrium, the macrophages increase in number in secretory phase; their physiological role is the clearance of cell debris over the course of the menstrual period. In eutopic endometrium of EM patients, this increase in macrophage number does not happen, but a global augmentation (hormonal cycle-independent) of macrophages (in particular of M1 macrophages) has been observed, as compared to the endometrium of non-EM women. In ectopic tissue, a high number of angiogenesis-supportive M2 macrophages are found in the lesions. The presence of M2 macrophages is predominant in lesions, peritoneal cavity and fluid of women with EM compared to healthy women (24, 25). Blood monocytes, once differentiated into tissue macrophages, increase proliferation of endometrial cells isolated from women affected by EM, whereas monocytes derived from healthy women inhibit endometrial cell proliferation (26).

### Uterine Natural Killer Cells

It is well known that uterine Natural Killer (uNK) cells, which are characterized by lower cytotoxicity (CD16^low^, CD56^bright^), increase in number during secretory and menstrual phases to establish a suitable environment for embryo implantation (27–29). In the uterine endometrium of EM patients, this fluctuation is maintained, although Giuliani et al. demonstrated a decreased uNK percentage and activity (30). The uNK cells present in the peritoneal fluid of women affected by EM showed a lower activity and, in the lesions, a lower capability to induce apoptosis of endometrial cells (31, 32). Subsequent studies confirmed a lower NK cell cytotoxic activity in EM; this reduction is associated with the severity of the disease (32).

### Mast Cells

An augmented number and activity of mast cells (MCs) is usually associated with normal endometrial tissue during menstruation; however, this remains debatable although MC role in tissue angiogenesis and regeneration is well established. A high numbers of degranulated MCs are a common characteristic of endometriotic lesions (33–38). The MC “fingerprint” of their involvement in acute inflammation is an increase in the production of secreted mediators such as pro-inflammatory cytokines (such as TNF-α); tryptase is currently considered one of the main diagnostic markers for MC activation (39). Borelli et al. demonstrated that peritoneal fluid of EM patients was tryptase enriched and could affect sperm motility (40).

The involvement of MCs in the EM lesion formation has been investigated in a recent study that showed that numerous MCs with heightened activation level were present in endometriotic lesions in both animal models and humans. MC stabilizers and inhibitors may be successfully used to treat EM. The high number of increasingly activated and degranulated MCs in deeply infiltrating EM and an intimate histological connection between MCs and nerves, indicate that MCs could play a pivotal role in the occurrence of pain and hyperalgesia in EM, most likely exerting a direct effect on nerve endings (37, 38).

In EM, an abundant infiltration of MCs can be detected around the stromal lesions. These MCs exhibit degranulation; scattered granules are also commonly identified. MCs are hardly observed within eutopic endometrium and normal uterine serosa of both EM patients and healthy women (41). MCs are present in endometrial cyst tissues. The localization of cells in the endometrial stroma is very limited, while many MCs can be seen around blood vessels and fibrotic interstitia, i.e., the fibrotic interstitium of endometrial cysts. Thus, MCs likely take part in the development of EM. Localization of MCs leads to a particularly strong association with adhesion and fibrosis (35, 36, 41).

### Eosinophils and Neutrophils

Although to a lesser extent compared to uNKs, eosinophil number increases normally during secretory and menstrual phases. A higher level of eotaxin (a chemoattractant for eosinophils), compared to normal endometrium, was described in both eutopic and ectopic endometrium of EM patients, as well as in peritoneal fluids of severe EM (42).

Neutrophil number increases only during the menstruation phase as well as in the endometrium of EM patient; but the neutrophils present in the eutopic tissue of EM patients, compared to those derived from healthy endometrium, present an increased activation state characterized by elevated reactive oxygen species production and CD11b expression (43, 44). The number of neutrophils are increased in the peritoneal cavity of EM patients; endometriotic neutrophils produce angiogenic factors and cytokines such as VEGF, IL-8, and CXCL10, and reactive oxygen species, which may support which may support the disease progression (45). In addition, Takamura et al. demonstrated a significant presence of neutrophils in ectopic endometrium, suggesting their role in the angiogenesis of the lesions (46).

### Lymphocytes

Aberrant T lymphocyte response to autologous endometrial cells has been observed in EM. Co-cultures of autologous lymphocytes and endometrial cells allowed evaluation of lymphocyte proliferation in response to autologous endometrium in controls and to ectopic and eutopic endometrial cells from patients. The lymphocyte proliferative response to autologous endometrial cells appeared to be lower in women with EM and in animal models of spontaneous EM (47, 48). Furthermore, employing a ^51^Cr microassay of lymphocytotoxicity to endometrial cell, Russel et al. demonstrated that T lymphocyte cytotoxicity against autologous endometrial cells is significantly decreased in women affected by EM (49–51). The defect in T-lymphocyte cytotoxicity, i.e. of CD8^+^ cytotoxic T cells, was resolved by recombinant IL-2 stimulation of peripheral blood lymphocytes (52).

Fas ligand (FasL) is able to induce lymphocyte apoptosis by binding to its receptor, Fas, which is also expressed on lymphocytes. Therefore, cells expressing high levels of FasL may induce apoptosis of surrounding lymphocytes, thereby preventing lymphocyte response. Remarkably, FasL expression in endometrial stromal cells is stimulated by IL-8, and CCL2, CCL12, and CCL13 cytokines/chemokines, which are also increased in the peritoneal fluids and sera of the EM patients. Soluble FasL, which also induces apoptosis in Fas-expressing cells, showed reduced levels in the peritoneal fluid of women with advanced stages of EM. The CD4:CD8 ratio appeared to be decreased in endometriotic peritoneal fluid. Although the total number of CD4^+^ T cells was found to be elevated, the activated status of CD4^+^ as well as CD8^+^ T cells, characterized by the expression of CD11, was decreased in endometriotic peritoneal fluid (49, 53).

The number of total and activated T lymphocytes is increased in ectopic endometrium in comparison with eutopic endometrium; IL-4 and IL-10 are upregulated in peripheral lymphocytes in women affected by EM. A higher IL-4 expression is also reported for lymphocytes present in endometriotic tissues and in peritoneal fluids. On the contrary, the production of IFN-γ is lower in peripheral lymphocytes in EM. T helper (Th)1/Th2 balance is shifted toward Th2 in EM (54). Hirata et al. recently demonstrated the presence of Th17 cells in peritoneal fluid of EM women. IL-17 stimulates EM stromal cell proliferation, and their expression of IL-8 and cyclooxygenase-2 (55). In eutopic endometrial tissues, the amount of regulatory T cells is significantly lower during the secretory phase in healthy women; this reduction was not present in EM women.

Studies have also focused on the role of B lymphocytes in the development of EM, considering in particular autoimmune responses. Wild and Shivers showed the presence of anti‐endometrial antibodies in the sera of EM patients (56). Anti--nuclear antibodies, anti-DNA antibodies, and anti-phospholipid antibodies have also been detected in women with EM and it is likely that Th2 polarization in EM is the precipitating factor for the appearance of these autoantibodies (57). The relationship between autoantibody and EM may also explain EM‐associated infertility, since these antibodies might bind not only to the endometrial tissue but also to embryos and sperms.

## Complement System

The complement system plays a very important role in the recognition and clearance of pathogens, apoptotic and necrotic cells (58–61).

The complement system is represented by over 50 proteins, including soluble activation precursor components, regulators and cell surface receptors (62). The complement system is very efficient at tagging or flagging the non-self (pathogens), altered self (apoptotic/necrotic cells, and protein aggregates), and transformed self (tumor cells), which can result in lysis of target cells/pathogens, opsonization and subsequent enhanced uptake by phagocytic cells of the immune system *via* complement receptors, and generation of inflammatory mediators. In addition, the complement system can also modulate the adaptive immune response, and act as a link between innate and adaptive immunity (63).

### Complement Classical Pathway

**Figure 2:**
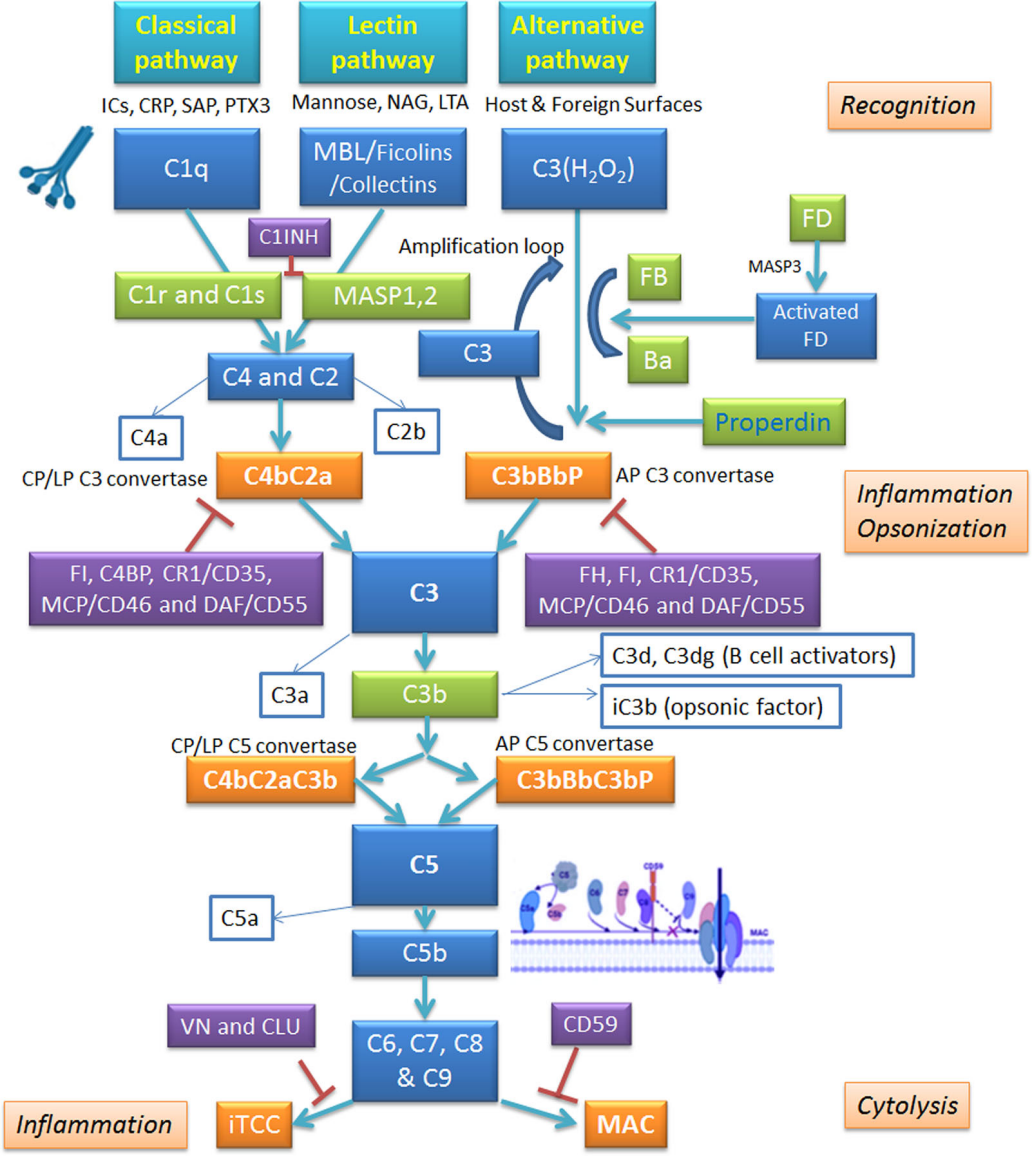
Schematic overview of the complement system and its regulators. The complement system operates *via* three pathways: classical, alternative and lectin. Classical pathway is triggered by binding of C1q to antigen-antibody complex; alternative pathway involves autoactivation of C3, whereas lectin pathway is set in motion by Mannan-Binding Lectin (MBL) interaction with carbohydrate patterns on pathogen surface. All pathways converge on C3 convertase; from there, they follow identical routes of the cascade. The complement activation is kept in check by inhibitory regulators. AP, alternative pathway; CLU, clusterin; CP, classical pathway; CRP, C-reactive protein; CR1, complement receptor 1; C1INH, C1 inhibitor; DAF, decay-accelerating factor; FB, factor B; FD, factor B; FH, factor H; FI, factor I; ICs, immunocomplexes; iTCC, inactive terminal C complex; LP, lectin pathway; LTA, lipoteichoic acid; MAC, membrane attack complex; MCP, membrane cofactor protein; NAG, N-acetylglucosamine; PTX3, pentraxin 3; SAP, serum amyloid P component; VN, vitronectin.

The complement system can be activated through three major pathways: classical, lectin, and alternative (**Figure 2**) (62, 64). The classical pathway is activated following the interaction of C1 complex with the antigen-antibody complex. C1 complex consists of three sub-components: C1q, C1r and C1s. Recognition of the Fc portion of cross-linked IgG1, IgG3 or IgM, fixed to a multivalent antigen, by C1q is the first step. The conformational change in the C1q molecule, induced by the bonding with immunoglobulins, activates the C1r subunit with serine-protease activity: this, in turn, triggers the proteolytic activity of the C1s molecule, which splits the subsequent protein of the complement cascade, the C4 molecule, in two fragments: C4a and C4b (65).The first fragment remains in circulation in the plasma, while the other binds covalently to membrane proteins and carbohydrates, ensuring that complement activity is maintained at a well-defined point. C4b, in the presence of Mg^2+^, binds the C2 molecule and makes it susceptible to cleavage by the C1s subunit; following hydrolysis, the two fragments C2a and C2b are yielded: C2a binds to C4b giving rise to the complex C4b2a. The enzyme C4b2a, better known as C3 convertase of the classical pathway, remains attached to the surface of the pathogen/target and hydrolyzes the molecule C3 into C3a and C3b (66). C3a (anaphylatoxin) is a potent inflammatory molecule; C3b opsonizes target pathogen and brings about phagocytosis by macrophages and polymorphonuclear cells. In addition, C3b interacts with C4b2a complex, yielding C5 convertase of the classical pathway (C4b2a3b) (66).

The C5 convertase cleaves the C5 molecule into C5a and C5b; C5b, by binding to C6, forms a hydrophilic complex, which undergoes a conformational change following association with C7. This favors the exposure of lipophilic groups with which the β subunit of the C8 molecule makes contact, while the α subunit penetrates the lipid double layer of the membrane of the target cell, following the conformational changes of the complex. Finally, the association of C5b678 with C9 induces its polymerization, and therefore, the formation of stable, cylinder-shaped transmembrane porous channels, which promote an osmotic imbalance, i.e. they alter the flow of ions and the gradient of molecules and water, inducing cell lysis. The final components of the complement system are designated as the membrane attack complex (MAC) (62, 64). Recent studies have established that the classic pathway can be activated, regardless of the presence of antibodies, by damage signal molecules such as C-reactive protein, viral proteins, amyloid β, polyanions (lipopolysaccharides, DNA and RNA), mitochondrial fragments, necrotic and apoptotic cells (60, 67).

### Complement Lectin Pathway

The lectin pathway is initiated by the binding of mannan-binding lectin (MBL), ficolin (1, 2 or 3), or collectin 11 (CL-K1) to mannose residues and other carbohydrate patterns present on the cell surface of pathogenic microorganisms (68, 69). The binding promotes the association of MBL with the serine proteases, MASP (MBL-Associated Serine Protease): there are three MASPs which form complexes with MBL oligomers, MASP-1 (70), MASP-2 (71), and MASP- 3 (72), and two non-enzymatic associated proteins, Map44 (73) and Map19 (74). MASP-1 and MASP-2 activate the lectin pathway of the complement system (75, 76) *via* cleavage of C4 and C2, while MASP-3 is responsible for the activation of alternative pathway (75, 77). The MASPs are close homologs of C1r and C1s but they form only dimers, not tetramers. C1 has a fixed stoichiometry of one C1q, two C1r, and two C1s. The C1r binds directly to the C1q, and C1s then binds to C1r, thus yielding a hetero-tetramer. It has become clear that the MBL-MASPs complexes are not quite equivalent to C1: they are smaller and very heterogeneous. MBL forms homodimeric complexes with MASPs. MASP activation, and C4 and C2 cleavage, leads to the formation of the C3 convertase of the lectin pathway or C4b2a. This enzyme cleaves the C3 molecule to C3a and C3b, the resulting C4b2a3b complex degrades C5 to C5a and C5b; the subsequent phases are similar to those of the classical pathway. This pathway seems to be active especially during childhood and during the transition period from passive immunity, operated by maternal antibodies, to active immunity (67).

### Complement Alternative Pathway

Unlike the classical and lectin pathways, the alternative pathway is independent of antigen-antibody complexes and can be directly induced by components of the cell wall of the bacteria and those present on the surface of the damaged host cells *via* C3. C3 consists of two polypeptide chains, α and β, linked by a disulfide bridge (78). Under normal physiological conditions, the C3 is subject to basal activation by spontaneous hydrolysis of its thioester residue (79). The product of the hydrolysis reaction is the C3 (H_2_O) molecule, which is rapidly inactivated in circulation; when bound to the surfaces of target cells, for example bacteria, it can associate with factor B (FB). As soon as it binds to C3 (H_2_O), FB loses a small fragment (Ba) by a protease called factor D (FD). The residual fragment, Bb, remains bound to C3 (H_2_O) making up the complex C3 (H_2_O) Bb. This enzyme complex is capable of splitting C3 into C3a and C3b, which analogously to C3 (H_2_O), binds the FB on which the FD acts, causing the excision of the fragment Ba and the formation of the enzyme complex C3bBb or C3 convertase of the alternative pathway (80). The C3 convertase of the alternative pathway is capable of splitting large quantities of C3, thus acting as a rapid amplification loop where fixed C3b molecules generated by either the classical or lectin pathway can bind to FB, resulting in FB cleavage by FD and generation of the convertase C3bBb (81). On pathogen surface, the highly labile C3 convertase is stabilized by factor P or properdin, increasing its half-life by 10-fold (82). Properdin is a plasma protein and the only known up-regulator of the alternative pathway. The next phase of this pathway is represented by the binding of the C3b molecule to the C3 convertase yielding C5 convertase, which cleaves the molecule C5 into C5a and C5b; the latter by binding to the C6–9 molecules participates in the formation of the MAC (83).

C3b is an intermediate that reacts with water, with the hydroxyl groups present on carbohydrates on the cell surfaces, with complexes of the immune system, and with free IgG, in a radius of about 60 nm from the point of its generation. Thanks to these interactions, the C3b molecule ensures its protection against inactivation by complement regulators, such as factor H (FH) and factor I (FI) (84); conversely, the free form in the fluid phase has a half-life of less than 1 s. It has also been noted that the C3b reacts preferentially with IgG, the second most abundant protein present in the plasma. The resulting complex, (C3b)2-IgG, seems to be the best precursor in the formation of C3 convertase of the alternative pathway, being less vulnerable to inactivation by FH (85). All this translates into greater effectiveness in the assembly of the convertases, involving properdin, which stabilizes the bond with FB and reduces the dissociation of the Bb fragment. The step described creates a amplification loop that theoretically could go on indefinitely, generating increasing quantities of the converted C3 and C3b molecule. The regulation of this positive feedback circuit depends on the concentration of FB and C3b molecules. The alternative pathway, in addition to carrying out the primary task of quickly covering the bacterial surface with high quantities of the opsonizing fragment of the complement, C3b, acts on the altered tissues of the host, characterized by cells that undergo apoptosis or at the site of wounds and infection (81).

### Regulators of Complement Activation

When an abnormal complement activation occurs, for instance in patients with dysfunctional regulatory proteins or affected by autoimmune pathologies, it can be responsible for a severe inflammatory response involving various organs (66). In some circumstances, C5b67 complexes can deposit on healthy neighboring host cells, causing their lysis. Clusterin, a regulatory molecule of the classical pathway, can bind to MAC and block the insertion of the complex in the membrane (86, 87). All host cells have CD59, a small 20kDa GPI-linked protein which binds C5b-8, and stops C9 binding. CD59 limits the incorporation of the C8 and C9 molecules, and therefore, the formation of the MAC (88). Furthermore, these complexes can elicit various metabolic and cellular pathways, in addition to the production of inflammatory mediators, such as prostaglandins and leukotrienes, inducing a diffuse inflammatory state. To ensure efficient regulation of the complement system, it is necessary to maintain the integrity of the regulatory proteins, since anomalies affecting them can lead to excessive activation of the complement and pathological states (66).

To overcome this, the host cells have defense systems to guarantee their own protection, i.e. soluble regulators in plasma or membrane-bound on their own cell surface. The first category includes the serine protease inhibitor C1-INH, responsible for inhibiting the C1 complex of the classical pathway and MASP-1 and MASP-2 of the lectin pathway (89); FI (90), the protagonist of the cleavage of the α chain of the molecules C3b, mediated by the FH; and C4b-binding protein (C4bp), which represents the main cofactor of FI in C4b degradation. Membrane-bound regulatory proteins include the cofactors of FI: Complement Receptor 1 (CR1, CD35) (91) and Decay Accelerating Factor (DAF; also designated as CD55) (92), which participate in C3b and C4b degradation, and Membrane Cofactor Protein (MCP; also called CD46) (93), which is involved in the prevention of the formation of the C3- and C5-convertases and the acceleration of their decay. When C5b-8 or C5b-9 assemble in plasma, several plasma proteins can bind to them, preventing its insertion into a lipid bilayer. These proteins include vitronectin and clusterin (alternative names: S-protein, SP40.40).

The surface of the host cells is also protected from the action of C3 convertase, thanks to polyanionic molecules exposed on the membrane such as glycosaminoglycans, heparin and sialic acid, which by binding to FH facilitate its interaction with C3b, promoting its hydrolysis by FI (94) Mutations affecting the genes coding for regulatory proteins, present in heterozygosity (haploinsufficiency), predispose subjects to pathologies, such as atypical Hemolytic Uremic Syndrome (aHUS), type II Membranoproliferative Glomerulonephritis and Macular Degeneration related to age (95).

## Complement System in The Endometrial Lesion

The presence of C3 in endometriotic tissue was first highlighted in 1980 by Weed and Arquembourg (96). Subsequently, Bartisik and co-workers (97) confirmed the presence of C3 and C4 in endometrial tissue of patients undergoing diagnostic laparoscopy. A number of studies have validated the presence of complement components in the EM lesions (98–103). Tao et al. estimated the gene expression of C3 distinguishing between human eutopic and ectopic endometrium; the expression of C3 mRNA and protein appeared to be significantly higher in human ectopic endometrium in comparison with matched eutopic one (104). Glandular epithelial cells present in endometriotic implants have been shown to secrete C3; its expression is up-regulated by estradiol (103, 105). A recent study showed an interesting association between a particular SNP involved in C3 gene upregulation and the increased risk for EM and EM-associated infertility (101). Despite evidence of the presence of complement components in EM, their contribution to EM pathogenesis is far from clear.

Endometrial tissue–specific complement activation is frequently observed in women affected by EM (105, 106). One of the likely causes of complement activation in the EM microenvironment is the triggering of the coagulation cascade due to periodic bleeding of EM tissue. Thrombin can be responsible for the cleavage of C3 to C3a and C3b; activated platelets are also implicated in C3 cleavage (107). Another activator of the C3 is heme that is released from hemoglobin during hemolysis: heme induces deposition of C3b on erythrocytes (108). Alternative pathway can be activated through properdin binding to activated platelets promoting C3 (H_2_O) recruitment and complement activation. In addition, the treatment of endothelial cells with C3a or other factors promptly stimulate the expression of P-selectin, which through the binding to C3b induces the formation of C3 convertases (109).

In EM lesions, the complement regulatory protein expression seems to be altered too. DAF expression levels were significantly reduced in samples isolated from EM patients during mid secretory phase. A decreased DAF protein level was confirmed in cells dissected by laser micro dissection (110).

## Complement Components in Endometriosis-Derived Cells

High-throughput studies have highlighted C3 and other complement genes as the most up-regulated genes in EM tissues in comparison with normal endometrium (98, 99, 111, 112). The gene expression profile of eutopic and ectopic endometrial stromal cells (98) revealed C3, C7 and SERPIN5 as highly expressed transcripts. Immortalized ectopic endometriotic stromal cells in EM have been shown to produce mainly C3 and PTX3 and display a differential regulation of iron metabolism (112).

In addition to estradiol, a potential factor for the upregulation of C3 expression by endometrial cells was the peritoneal fluid rich in pro-inflammatory factors (113); TNF-α and IL-1β levels appeared to be increased in the peritoneal fluid of patients with EM, and higher levels of TNF-α seemed to be associated with more advanced stages of the disease (113–116).

Studies by Suryawanshi et al. (99) and Edwards et al. (117) revealed that chronic inflammation in EM is dominated by the complement system, which remains active in EM-associated ovarian cancer (EAOC) but not tumors, further demonstrating heterogeneity in the inflammatory *milieu* within ovarian cancer. C7, FD, FB, FH, and MASP1 are differentially expressed in EM compared to normal tissue. They observed an increase in C7, FD, FB, FH and a decrease in MASP-1 levels. Furthermore, C3 and C4A were up-regulated in EM compared to normal tissues as well as EAOC (99).

It is clear that further investigation is required to identify the factors that are produced by the endometrial tissue disseminated within the abdominal cavity which impact on the release of C3 and other components by the uterine endometrium (102). Although the pertinent role of the complement system in EM has been repeatedly confirmed, studies using complement gene knock-out animals, offering important insights into the pathogenesis and therapeutic interventions have been missing. We have recently generated a murine model of EM *via* injection of minced uterine tissue from a donor mouse into the recipient mice peritoneum using wild type and C3 gene-deficient (C3^-/-^) mice (118). We found that the C3-deficient mice showed a lower amount of EM cyst formation in the peritoneum than the wild-type mice. Furthermore, peritoneal washing from the wild type mice with EM showed more degranulated MCs compared to C3^-/-^ mice, consistent with higher C3a levels in the peritoneal fluid of EM patients. Thus, C3a participates in an auto-amplifying loop, leading to MCs infiltration and activation, which is pathogenic in EM (118).

## Complement in Endometriosis Peritoneal Fluid

Complement activation can generate C3a and C5a, two well-known anaphylatoxins, which are capable of stimulating the peritoneal MCs and macrophages to produce mediators such as histamine or cytokines, which in turn increases the endometrial vascular permeability (102), causing inflammation and pain. We observed increased levels of C3a in the peritoneal fluid of EM patients (118); Kabut et al. (119) have also reported increased levels of C3c, C4, and sC5b-9 in the peritoneal fluid and serum of EM patients in comparison with healthy women. A recent study reported significantly higher concentrations of C1q, MBL and C1-INH in the peritoneal fluids of EM women as compared to the control group (120). No difference in plasma C3a levels between women with and without EM was found (121).

## Possibility of Anti-Complement Immunotherapy in Endometriosis

All currently available treatments for EM are not curative but suppressive and are accountable just for a transitory relief of the symptoms. The prevalent therapeutic options for relieving EM-associated pain are represented by contraceptive rather than fertility-promoting treatments (122). Immunotherapy is beginning to be considered as an option for EM treatment.

**Figure 3:**
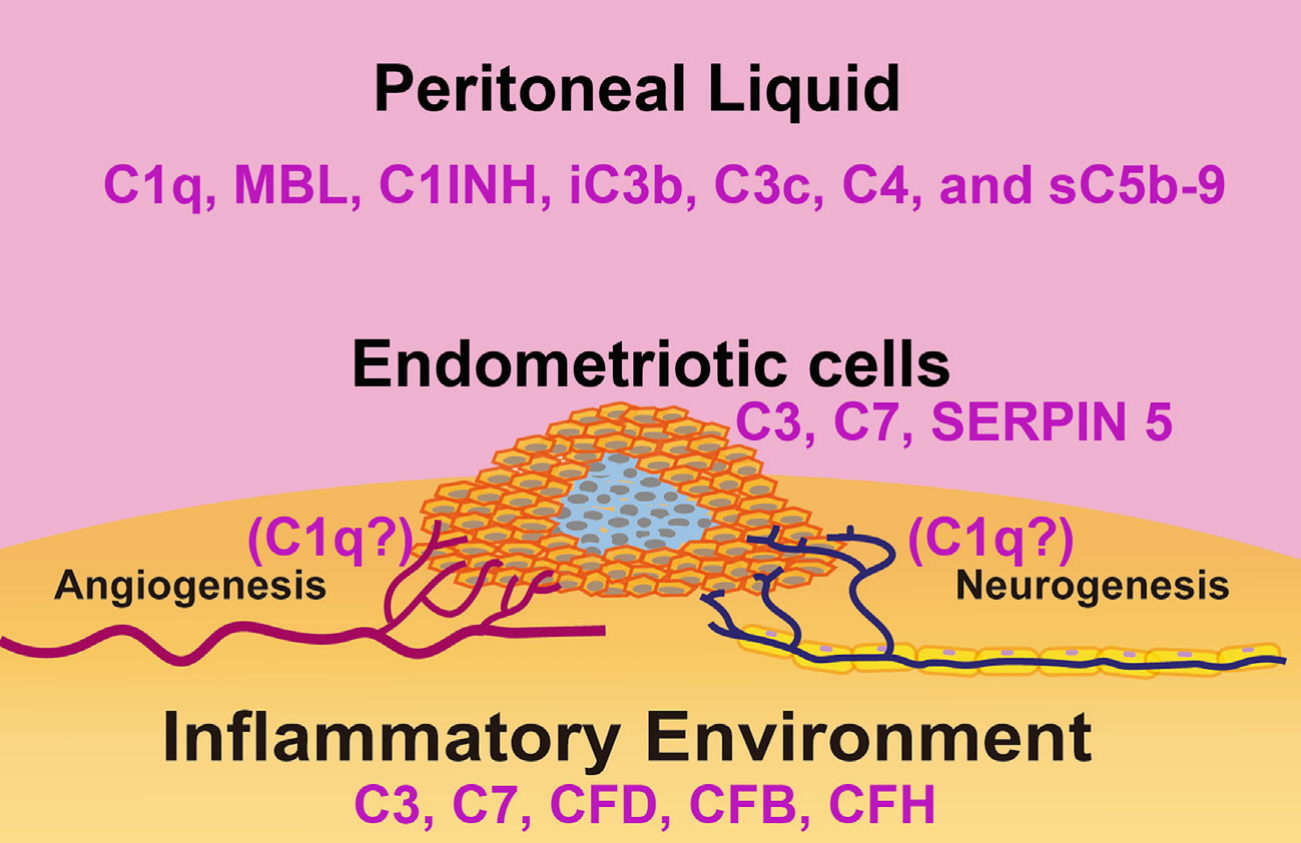
Schematic representation of the complement system in the immune microenvironment of an endometriotic lesion. The lesions consist of epithelial, endothelial, stromal cells and leukocytes present in the tissue and surrounded by peritoneal liquid. Studies have linked disorders of the complement system activity/expression and pathogenesis of endometriosis. In the endometriotic lesions, high levels of C3, C7, factor D (FD), factor B (FB), and factor H (FH) have been detected. Isolated endometriotic cells express C3, C7 and SERPIN5. In the peritoneal liquid of EM patients, elevated levels of C1q, C4, Mannan-Binding Lectin (MBL), C1 Inhibitor (C1INH), inactivated C3b (iC3b), C3c and soluble C5b-9 (sC5b-9) have been reported.

The first drug that blocked the complement pathway and approved for clinical trials was Eculizumab; the other drug currently approved is used for the treatment of hereditary angioedema (HAE): C1INH (Berinert, Cinryze, Ruconest) (123, 124) (**Figure 3**). Food and Drug Administration (FDA) approved Eculizumab (anti-C5 antibody) for the treatment of paroxysmal nocturnal hemoglobinuria (PNH) in 2007 (125), 40 years after the demonstration that complement was the principal cause of this devastating disease (126, 127). The clinical use of eculizumab for PNH therapy demonstrated that blocking of complement could be relatively safe, bringing life-changing results (128).

FDA approval of eculizumab for treating the rare renal disease aHUS in 2011 has fueled development of new anti-complement drugs for clinical trials over the last few years (129–132); some have entered clinical development while others are in phase 3 trials. The use of Eculizumab may be promising in the treatment of EM, although blocking the complement activation at C5 level could leave uncovered all the effects induced by C3 activation (e.g. C3a formation), a pivotal step in the EM pathogenesis.

Most efforts to improve complement-targeting immunotherapy are aimed at improving the pharmacokinetic properties of these drugs: to reduce the dose. A next-generation “recycling” form of eculizumab, called ravulizumab (Ultomiris™) (133), has been approved by both FDA and European Medicines Agency for the treatment of PNH, and is currently under review for aHUS (Ultomiris™). “Recycling” or “pH-switched” antibodies are produced by changing the antigen-binding region (incorporating histidine residues) of existing antibodies, so that the antibody loses affinity in the acidic pH 6.0 environment of the endosome. After the internalization of the antibody into the cells, the acidic pH causes the release of the target and recycling of the “empty” antibody back to the circulation (134). Crovalimab (Roche; SKY59) is another recycling anti-C5 antibody that is currently in phase II clinical trial (135–137).

An additional approach to reduce drug dose is to develop an antibody that binds neoepitopes on complement proteins rather than using one directed against the native protein. Several drugs are currently undergoing clinical evaluation, such as IFX-1 (InflaRx), which targets the released C5a fragment (138), and BIVV020 (Sanofi), a preclinical antibody developed for binding to activated C1s. It is largely expected that these anti-complement drugs can be of great potential in setting up clinical trials in EM patients.

The current pandemic caused by severe acute respiratory syndrome coronavirus 2 (SARS-CoV-2) infection has led to the testing of some complement immunotherapy drugs as well. Compstatin-based C3 inhibitor AMY-101 was safely and successfully used for the treatment of a patient with COVID-19 pneumonia (139, 140). Narsoplimab, a lectin-pathway inhibitor, has been shown to prevent the initiation of the lectin pathway and endothelial cell damage induced by SARS-CoV-2, lowering the risk of thrombotic disorders; all patients who received narsoplimab treatment recovered and survived without exhibiting any drug-related adverse effect (141).

**Figure 4:**
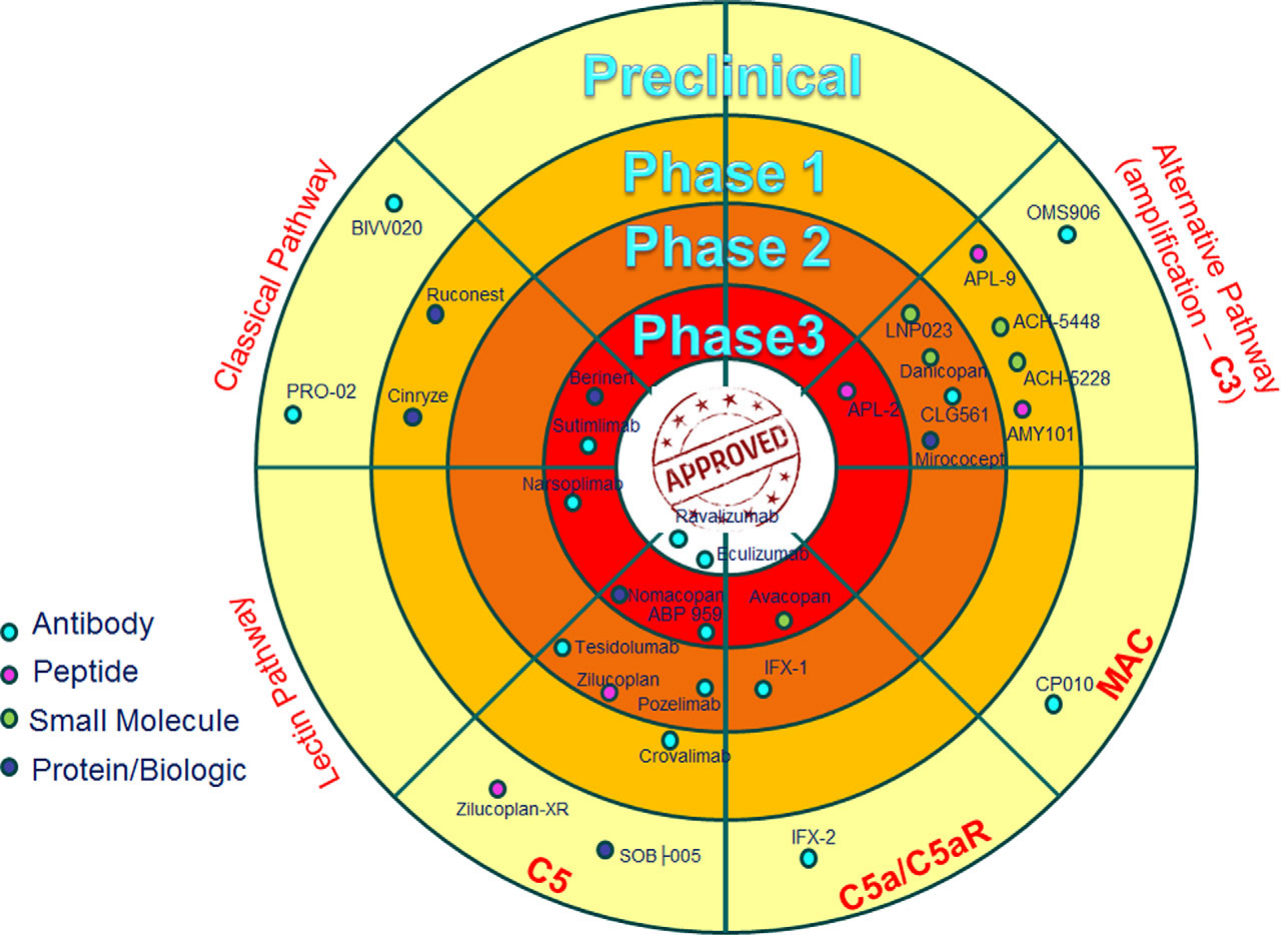
Anti-complement drugs currently in clinical development. The dartboard with concentric rings indicates the different phases of clinical development, with “approved” in the centre. Only drugs currently in clinical development are shown and the most advanced stage of development for any indication is shown. The drugs are divided on the basis of the target (Classical, Lectin and Alternative Pathways), with the exception of some drugs that are directed to the common part of the three cascades (C5 and MAC), or drugs that are specifically blocking C5a activity (C5a/C5aR) (128).

AMY-101 (and other C3 inhibitors) could be a promising treatment for relapsing EM as well, being able to block C3 activation (128, 142–145). It is anticipated that a MASP inhibitor such as Narsoplimab will not exert a massive therapeutic effect on the EM pathogenesis since no evidence is available so far that is suggestive of a key involvement of the complement lectin pathway in the EM lesion formation. Various prospective anti-complement inhibitors studied so far are listed in **Table 1** and **Figure 4** (128, 141–176).

**Table 1:** Principal anti-complement drugs.

Drug name	Target	References
ABP 959	C5	Chow et al. (146)
ACH-5448	FD	Zelek et al. (128)
ACH-5228	FD	Zelek et al. (128)
AMY101	C3	Mastellos et al. (142)
APL-2	C3	Zelek et al. (128); Wong et al. (143); Grossi et al. (144); Grossi et al. (145)
APL-9	C3	Zelek et al. (128)
AVACOPAN	C5aR1	Merkel et al. (147); Tesar et al. (148); Jayne et al. (149); Bekker et al. (150)
BERINERT	C1r/C1s, MASPs	Keating (151); Zanichelli et al. (152)
BIVV020	activated C1s	Zelek et al. (128)
CINRYZE	C1r/C1s, MASPs	Aygören-Pürsün et al. (153); Lyseng-Williamson (154); Bernstein (155)
CLG561	Properdin	Zelek et al. (128)
CP010	C6	Zelek et al. (128)
CROVALIMAB	C5	Röth et al. (156)
DANICOPAN	FD	Wiles et al. (157); Risitano et al. (158)
ECULIZUMAB	C5	Zelek et al. (128); Wijnsma et al. (159)
IFX-1	C5a	Zelek et al. (128); Giamarellos-Bourboulis et al. (160); Vlaar et al. (161)
IFX-2	C5a	Zelek et al. (128)
LNP023	FB	Zelek et al. (128)
MIROCOCEPT	CR1	Halstead et al. (162); Kassimatis et al. (163)
NOMACOPAN	C5	Schols et al. (164)
NARSOPLIMAB	MASP2	Rambaldi et al. (141); Elhadad et al. (165); Selvaskandan et al. (166)
OMS906	MASP3	Zelek et al. (128)
POZELIMAB	C5	Latuszek et al. (167)
PRO-02	C2	Zelek et al. (128)
RUCONEST	C1r/C1s, MASPs	Busse et al. (168); Cruz (169)
SOB├005	C5	Zelek et al. (128)
SUTIMLIMAB	C1s	Freire et al. (170); Nikitin et al. (171); Bartko et al. (172); Jäger et al. (173)
TESIDOLUMAB	C5	Jordan et al. (174)
ZILUCOPLAN	C5	Albazli et al. (175); Wilkinson et al. (176)
ZILUCOPLAN-XR	C5	Zelek et al. (128)

## Endometriosis and Immunotherapy

We have discussed a strong link between EM and alterations of local and systemic immune system. In the context of EM, NK cells may be exploited as a potential target for immunotherapy. A reduced NK cell activity in women with EM was first reported by Oosterlynck et al. (177); a lowered NK cell cytotoxic action against autologous endometrial cells, both in peripheral blood and peritoneal fluid, was noted correlating with the severity of the disease (177, 178). In fact, EM is characterized by a downregulation of NK cell cytotoxicity (179, 180), probably due to the consistent amount of inhibitory cytokines in the peritoneal fluid of patients affected by EM, or to an augmented presence of several inhibitory NK cell receptors. It is reasonable to speculate that the incapacity of uNK cells, as well as macrophages, to recognize and eliminate endometriotic cells in the peritoneal cavity, can allow their survival and growth, leading to development and progression of EM (181).

In cancer, the activity of NK cells may depend on checkpoint molecules (182, 183). Thus, in EM, the activation of some checkpoint molecules could be involved in the reduced elimination of shed endometrial cells. For this reason, EM patients may benefit from suppressing NK cell negative control checkpoints, such as inhibitory NK cell receptors. Understanding checkpoints involved in the downregulation of NK cell activity in the progression of EM may be important for identifying new therapeutic targets (181).

A possible interaction between complement and impaired NK cell function has been demonstrated (184). In fact, Liu et al. hypothesized a role for the complement receptor, CR3, in imbalancing the tumor surveillance function of NK cells and suggested that the iC3b/CR3 signaling is a pivotal negative mediator of NK cell activity (184). Considering the consistent expression of CR3 on NK cells (185), negative regulatory roles of iC3b/CR3 axis and high level of iC3b in the peritoneal fluid of EM women (119), we can assume that iC3b/CR3 signaling is an important negative regulator of uNK cell function in EM, which possibly exerts a negative influence on cytotoxicity against autologous endometrial cells. Although high concentrations of C3c, C4, and sC5b-9 were found in the serum of patients with EM, iC3b levels were higher in the peritoneal fluid (119).

We consider that a complement C3 inhibitor could be used as a treatment for EM, exerting two potentially beneficial effects: on the one hand, one would attain an interruption from the beginning of the cascade of inflammatory signals that plays an important role in the pathogenesis of EM; on the other hand, a lower production of iC3b would result in a lower inhibition of the cytotoxic activity of the NK cells. The activation induced by C5a and C3a of macrophages and MCs in the endometriotic microenvironment could be partially blocked (or considerably reduced) by complement immune therapy; for instance, Danicopan could act to stop the auto-amplifying loop induced by C3a on MCs (118). Furthermore, C1q, present at high levels in the peritoneal fluid of the EM patients, can induce the differentiation of the tissue macrophages towards M2 phenotype (186, 187), and hence, promote the angiogenesis in EM lesions (188). Thus, an antibody able to block specifically the alternative functions of C1q could be interesting to test in the EM treatment, as well as in tumor development (189, 190).

In conclusion, EM is a devastating disease that has a range of social, personal and medical consequences for the women suffering from it. It has become apparent that immune dysregulation is a major factor that precipitates this pathological condition. While immune infiltration and constant inflammatory milieu foster the disease, the biggest pathological consequences arise from aberrant complement activation. There are a number of ways to suppress complement activation in EM patients. This therapeutic intervention needs careful pre-clinical and clinical trials, involving monotherapy or in combination with checkpoint inhibitors.

## Author Contributions

CA, AB, AM, GZ, FR, GR, UK, and RB reviewed the literature and wrote sections of the review article. CA created figures. UK critically reviewed the entire manuscript. All authors contributed to the article and approved the submitted version.

## Funding

The research was funded by the Italian Ministry of Health (RC 20/16, RC 23/18, RC 24/19 to GR and 5MILLE15D to CA – Institute for Maternal and Child Health IRCCS Burlo Garofolo, Trieste, Italy).

## Conflict of Interest

The authors declare that the research was conducted in the absence of any commercial or financial relationships that could be construed as a potential conflict of interest.

## References

[1] Zondervan KT Becker CM Koga K Missmer SA Taylor RN Vigano P Endometriosis. Nat Rev Dis Primers (2018) 4(1):9. 10.1038/s41572-018-0008-5 30026507

[2] Zondervan KT Becker CM Missmer SA Endometriosis. N Engl J Med (2020) 382(13):1244–56. 10.1056/NEJMra1810764 32212520

[3] Acien P Velasco I Endometriosis: a disease that remains enigmatic. ISRN Obstet Gynecol (2013) 2013:242149. 10.1155/2013/242149 23956867 PMC3730176

[4] Shafrir AL Farland LV Shah DK Harris HR Kvaskoff M Zondervan K Risk for and consequences of endometriosis: A critical epidemiologic review. Best Pract Res Clin Obstet Gynaecol (2018) 51:1–15. 10.1016/j.bpobgyn.2018.06.001 30017581

[5] Stuparich MA Donnellan NM Sanfilippo JS Endometriosis in the Adolescent Patient. Semin Reprod Med (2017) 35(1):102–9. 10.1055/s-0036-1597121 27992932

[6] Rolla E Endometriosis: advances and controversies in classification, pathogenesis, diagnosis, and treatment. F1000Res (2019) 8(F1000 Faculty Rev):529. 10.12688/f1000research.14817.1 PMC6480968 31069056

[7] Agarwal N Subramanian A Endometriosis - morphology, clinical presentations and molecular pathology. J Lab Phys (2010) 2(1):1–9. 10.4103/0974-2727.66699 PMC3147077 21814398

[8] Mehedintu C Plotogea MN Ionescu S Antonovici M Endometriosis still a challenge. J Med Life (2014) 7(3):349–57. PMC4233437 25408753

[9] Wang Y Nicholes K Shih IM The Origin and Pathogenesis of Endometriosis. Annu Rev Pathol (2020) 15:71–95. 10.1146/annurev-pathmechdis-012419-032654 31479615 PMC7980953

[10] Dyson MT Bulun SE Cutting SRC-1 down to size in endometriosis. Nat Med (2012) 18(7):1016–8. 10.1038/nm.2855 PMC4077338 22772552

[11] Lagana AS Garzon S Gotte M Vigano P Franchi M Ghezzi F The Pathogenesis of Endometriosis: Molecular and Cell Biology Insights. Int J Mol Sci (2019) 20(22):5615. 10.3390/ijms20225615 PMC6888544 31717614

[12] Liu H Lang JH Is abnormal eutopic endometrium the cause of endometriosis? The role of eutopic endometrium in pathogenesis of endometriosis. Med Sci Monit (2011) 17(4):RA92–9. 10.12659/msm.881707 PMC3539524 21455119

[13] Li F Alderman MH 3rd Tal A Mamillapalli R Coolidge A Hufnagel D Hematogenous Dissemination of Mesenchymal Stem Cells from Endometriosis. Stem Cells (2018) 36(6):881–90. 10.1002/stem.2804 PMC5992028 29450941

[14] Hey-Cunningham AJ Fazleabas AT Braundmeier AG Markham R Fraser IS Berbic M Endometrial stromal cells and immune cell populations within lymph nodes in a nonhuman primate model of endometriosis. Reprod Sci (2011) 18(8):747–54. 10.1177/1933719110397210 PMC4051399 21617251

[15] Jerman LF Hey-Cunningham AJ The role of the lymphatic system in endometriosis: a comprehensive review of the literature. Biol Reprod (2015) 92(3):64. 10.1095/biolreprod.114.124313 25588508

[16] Suginami H A reappraisal of the coelomic metaplasia theory by reviewing endometriosis occurring in unusual sites and instances. Am J Obstet Gynecol (1991) 165(1):214–8. 10.1016/0002-9378(91)90254-o 1853899

[17] Polyzos NP Mauri D Tsioras S Messini CI Valachis A Messinis IE Intraperitoneal dissemination of endometrial cancer cells after hysteroscopy: a systematic review and meta-analysis. Int J Gynecol Cancer (2010) 20(2):261–7. 10.1111/igc.0b013e3181ca2290 20169669

[18] Pathan ZA Dinesh U Rao R Scar endometriosis. J Cytol (2010) 27(3):106–8. 10.4103/0970-9371.71877 PMC2983076 21187878

[19] Herington JL Bruner-Tran KL Lucas JA Osteen KG Immune interactions in endometriosis. Expert Rev Clin Immunol (2011) 7(5):611–26. 10.1586/eci.11.53 PMC3204940 21895474

[20] Symons LK Miller JE Kay VR Marks RM Liblik K Koti M The Immunopathophysiology of Endometriosis. Trends Mol Med (2018) 24(9):748–62. 10.1016/j.molmed.2018.07.004 30054239

[21] Koninckx PR Ussia A Adamyan L Wattiez A Gomel V Martin DC Pathogenesis of endometriosis: the genetic/epigenetic theory. Fertil Steril (2019) 111(2):327–40. 10.1016/j.fertnstert.2018.10.013 30527836

[22] Miller JE Ahn SH Monsanto SP Khalaj K Koti M Tayade C Implications of immune dysfunction on endometriosis associated infertility. Oncotarget (2017) 8(4):7138–47. 10.18632/oncotarget.12577 PMC5351695 27740937

[23] Ahn SH Monsanto SP Miller C Singh SS Thomas R Tayade C Pathophysiology and Immune Dysfunction in Endometriosis. BioMed Res Int (2015) 2015:795976. 10.1155/2015/795976 26247027 PMC4515278

[24] Bacci M Capobianco A Monno A Cottone L Di Puppo F Camisa B Macrophages are alternatively activated in patients with endometriosis and required for growth and vascularization of lesions in a mouse model of disease. Am J Pathol (2009) 175(2):547–56. 10.2353/ajpath.2009.081011 PMC2716955 19574425

[25] Khan KN Masuzaki H Fujishita A Kitajima M Sekine I Ishimaru T Higher activity by opaque endometriotic lesions than nonopaque lesions. Acta Obstet Gynecol Scand (2004) 83(4):375–82. 10.1111/j.0001-6349.2004.00229.x 15005786

[26] Braun DP Muriana A Gebel H Rotman C Rana N Dmowski WP Monocyte-mediated enhancement of endometrial cell proliferation in women with endometriosis. Fertil Steril (1994) 61(1):78–84. 10.1016/s0015-0282(16)56456-5 8293848

[27] Stabile H Carlino C Mazza C Giliani S Morrone S Notarangelo LD Impaired NK-cell migration in WAS/XLT patients: role of Cdc42/WASp pathway in the control of chemokine-induced beta2 integrin high-affinity state. Blood (2010) 115(14):2818–26. 10.1182/blood-2009-07-235804 PMC2854428 20130240

[28] King AE Critchley HO Oestrogen and progesterone regulation of inflammatory processes in the human endometrium. J Steroid Biochem Mol Biol (2010) 120(2-3):116–26. 10.1016/j.jsbmb.2010.01.003 20067835

[29] King A Uterine leukocytes and decidualization. Hum Reprod Update (2000) 6(1):28–36. 10.1093/humupd/6.1.28 10711827

[30] Giuliani E Parkin KL Lessey BA Young SL Fazleabas AT Characterization of uterine NK cells in women with infertility or recurrent pregnancy loss and associated endometriosis. Am J Reprod Immunol (2014) 72(3):262–9. 10.1111/aji.12259 PMC4126872 24807109

[31] Thiruchelvam U Wingfield M O’Farrelly C Increased uNK Progenitor Cells in Women With Endometriosis and Infertility are Associated With Low Levels of Endometrial Stem Cell Factor. Am J Reprod Immunol (2016) 75(4):493–502. 10.1111/aji.12486 26791471

[32] Kikuchi Y Ishikawa N Hirata J Imaizumi E Sasa H Nagata I Changes of peripheral blood lymphocyte subsets before and after operation of patients with endometriosis. Acta Obstet Gynecol Scand (1993) 72(3):157–61. 10.3109/00016349309013364 8385848

[33] Kempuraj D Papadopoulou N Stanford EJ Christodoulou S Madhappan B Sant GR Increased numbers of activated mast cells in endometriosis lesions positive for corticotropin-releasing hormone and urocortin. Am J Reprod Immunol (2004) 52(4):267–75. 10.1111/j.1600-0897.2004.00224.x 15494048

[34] Konno R Yamada-Okabe H Fujiwara H Uchiide I Shibahara H Ohwada M Role of immunoreactions and mast cells in pathogenesis of human endometriosis–morphologic study and gene expression analysis. Hum Cell (2003) 16(3):141–9. 10.1111/j.1749-0774.2003.tb00146.x 15005245

[35] Sugamata M Ihara T Uchiide I Increase of activated mast cells in human endometriosis. Am J Reprod Immunol (2005) 53(3):120–5. 10.1111/j.1600-0897.2005.00254.x 15727565

[36] Anaf V Chapron C El Nakadi I De Moor V Simonart T Noel JC Pain, mast cells, and nerves in peritoneal, ovarian, and deep infiltrating endometriosis. Fertil Steril (2006) 86(5):1336–43. 10.1016/j.fertnstert.2006.03.057 17007852

[37] Kirchhoff D Kaulfuss S Fuhrmann U Maurer M Zollner TM Mast cells in endometriosis: guilty or innocent bystanders? Expert Opin Ther Targets (2012) 16(3):237–41. 10.1517/14728222.2012.661415 22332753

[38] Paula R Jr Oliani AH Vaz-Oliani DC D’Avila SC Oliani SM Gil CD The intricate role of mast cell proteases and the annexin A1-FPR1 system in abdominal wall endometriosis. J Mol Histol (2015) 46(1):33–43. 10.1007/s10735-014-9595-y 25201101

[39] Butterfield JH Ravi A Pongdee T Mast Cell Mediators of Significance in Clinical Practice in Mastocytosis. Immunol Allergy Clin North Am (2018) 38(3):397–410. 10.1016/j.iac.2018.04.011 30007459

[40] Borelli V Martinelli M Luppi S Vita F Romano F Fanfani F Mast Cells in Peritoneal Fluid From Women With Endometriosis and Their Possible Role in Modulating Sperm Function. Front Physiol (2019) 10:1543. 10.3389/fphys.2019.01543 31998139 PMC6964357

[41] Matsuzaki S Canis M Darcha C Fukaya T Yajima A Bruhat MA Increased mast cell density in peritoneal endometriosis compared with eutopic endometrium with endometriosis. Am J Reprod Immunol (1998) 40(4):291–4. 10.1111/j.1600-0897.1998.tb00420.x 9784802

[42] Hornung D Dohrn K Sotlar K Greb RR Wallwiener D Kiesel L Localization in tissues and secretion of eotaxin by cells from normal endometrium and endometriosis. J Clin Endocrinol Metab (2000) 85(7):2604–8. 10.1210/jcem.85.7.6665 10902814

[43] Arici A Local cytokines in endometrial tissue: the role of interleukin-8 in the pathogenesis of endometriosis. Ann N Y Acad Sci (2002) 955:101–9. 10.1111/j.1749-6632.2002.tb02770.x 11949939

[44] Takehara M Ueda M Yamashita Y Terai Y Hung YC Ueki M Vascular endothelial growth factor A and C gene expression in endometriosis. Hum Pathol (2004) 35(11):1369–75. 10.1016/j.humpath.2004.07.020 15668894

[45] Izumi G Koga K Takamura M Makabe T Satake E Takeuchi A Involvement of immune cells in the pathogenesis of endometriosis. J Obstet Gynaecol Res (2018) 44(2):191–8. 10.1111/jog.13559 29316073

[46] Takamura M Koga K Izumi G Urata Y Nagai M Hasegawa A Neutrophil depletion reduces endometriotic lesion formation in mice. Am J Reprod Immunol (2016) 76(3):193–8. 10.1111/aji.12540 27432477

[47] Helvacioglu A Aksel S Peterson RD Endometriosis and autologous lymphocyte activation by endometrial cells. Are lymphocytes or endometrial cell defects responsible? J Reprod Med (1997) 42(2):71–5. 9058340

[48] Dmowski WP Steele RW Baker GF Deficient cellular immunity in endometriosis. Am J Obstet Gynecol (1981) 141(4):377–83. 10.1016/0002-9378(81)90598-6 7282821

[49] Osuga Y Koga K Hirota Y Hirata T Yoshino O Taketani Y Lymphocytes in endometriosis. Am J Reprod Immunol (2011) 65(1):1–10. 10.1111/j.1600-0897.2010.00887.x 20584009

[50] Gilmore SM Aksel S Hoff C Peterson RD In vitro lymphocyte activity in women with endometriosis–an altered immune response? Fertil Steril (1992) 58(6):1148–52. 10.1016/S0015-0282(16)55560-5 1459264

[51] Steele RW Dmowski WP Marmer DJ Immunologic aspects of human endometriosis. Am J Reprod Immunol (1984) 6(1):33–6. 10.1111/j.1600-0897.1984.tb00106.x 6476182

[52] Melioli G Semino C Semino A Venturini PL Ragni N Recombinant interleukin-2 corrects in vitro the immunological defect of endometriosis. Am J Reprod Immunol (1993) 30(4):218–27. 10.1111/j.1600-0897.1993.tb00623.x 7907480

[53] Oosterlynck DJ Meuleman C Lacquet FA Waer M Koninckx PR Flow cytometry analysis of lymphocyte subpopulations in peritoneal fluid of women with endometriosis. Am J Reprod Immunol (1994) 31(1):25–31. 10.1111/j.1600-0897.1994.tb00843.x 8166944

[54] Podgaec S Abrao MS Dias JA Jr Rizzo LV de Oliveira RM Baracat EC Endometriosis: an inflammatory disease with a Th2 immune response component. Hum Reprod (2007) 22(5):1373–9. 10.1093/humrep/del516 17234676

[55] Hirata T Osuga Y Hamasaki K Yoshino O Ito M Hasegawa A Interleukin (IL)-17A stimulates IL-8 secretion, cyclooxygensase-2 expression, and cell proliferation of endometriotic stromal cells. Endocrinology (2008) 149(3):1260–7. 10.1210/en.2007-0749 18079209

[56] Wild RA Shivers CA Antiendometrial antibodies in patients with endometriosis. Am J Reprod Immunol Microbiol (1985) 8(3):84–6. 10.1111/j.1600-0897.1985.tb00314.x 3895994

[57] de Barros IBL Malvezzi H Gueuvoghlanian-Silva BY Piccinato CA Rizzo LV Podgaec S “What do we know about regulatory T cells and endometriosis? A systematic review”. J Reprod Immunol (2017) 120:48–55. 10.1016/j.jri.2017.04.003 28463710

[58] Daha MR Role of complement in innate immunity and infections. Crit Rev Immunol (2010) 30(1):47–52. 10.1615/critrevimmunol.v30.i1.30 20370619

[59] Conigliaro P Triggianese P Ballanti E Perricone C Perricone R Chimenti MS Complement, infection, and autoimmunity. Curr Opin Rheumatol (2019) 31(5):532–41. 10.1097/BOR.0000000000000633 31192812

[60] Trouw LA Blom AM Gasque P Role of complement and complement regulators in the removal of apoptotic cells. Mol Immunol (2008) 45(5):1199–207. 10.1016/j.molimm.2007.09.008 17961651

[61] Dunkelberger JR Song WC Complement and its role in innate and adaptive immune responses. Cell Res (2010) 20(1):34–50. 10.1038/cr.2009.139 20010915

[62] Walport MJ Complement. First of two parts. N Engl J Med (2001) 344(14):1058–66. 10.1056/NEJM200104053441406 11287977

[63] Hajishengallis G Reis ES Mastellos DC Ricklin D Lambris JD Novel mechanisms and functions of complement. Nat Immunol (2017) 18(12):1288–98. 10.1038/ni.3858 PMC5706779 29144501

[64] Walport MJ Complement. Second of two parts. N Engl J Med (2001) 344(15):1140–4. 10.1056/NEJM200104123441506 11297706

[65] Lu J Kishore U C1 Complex: An Adaptable Proteolytic Module for Complement and Non-Complement Functions. Front Immunol (2017) 8:592. 10.3389/fimmu.2017.00592 28596769 PMC5442170

[66] Noris M Remuzzi G Overview of complement activation and regulation. Semin Nephrol (2013) 33(6):479–92. 10.1016/j.semnephrol.2013.08.001 PMC3820029 24161035

[67] Ehrnthaller C Ignatius A Gebhard F Huber-Lang M New insights of an old defense system: structure, function, and clinical relevance of the complement system. Mol Med (2011) 17(3-4):317–29. 10.2119/molmed.2010.00149 PMC3060978 21046060

[68] Degn SE Jensenius JC Bjerre M The lectin pathway and its implications in coagulation, infections and auto-immunity. Curr Opin Organ Transplant (2011) 16(1):21–7. 10.1097/MOT.0b013e32834253df 21150610

[69] Beltrame MH Catarino SJ Goeldner I Boldt AB de Messias-Reason IJ The lectin pathway of complement and rheumatic heart disease. Front Pediatr (2014) 2:148. 10.3389/fped.2014.00148 25654073 PMC4300866

[70] Matsushita M Fujita T Activation of the classical complement pathway by mannose-binding protein in association with a novel C1s-like serine protease. J Exp Med (1992) 176(6):1497–502. 10.1084/jem.176.6.1497 PMC2119445 1460414

[71] Thiel S Vorup-Jensen T Stover CM Schwaeble W Laursen SB Poulsen K A second serine protease associated with mannan-binding lectin that activates complement. Nature (1997) 386(6624):506–10. 10.1038/386506a0 9087411

[72] Dahl MR Thiel S Matsushita M Fujita T Willis AC Christensen T MASP-3 and its association with distinct complexes of the mannan-binding lectin complement activation pathway. Immunity (2001) 15(1):127–35. 10.1016/s1074-7613(01)00161-3 11485744

[73] Degn SE Hansen AG Steffensen R Jacobsen C Jensenius JC Thiel S MAp44, a human protein associated with pattern recognition molecules of the complement system and regulating the lectin pathway of complement activation. J Immunol (2009) 183(11):7371–8. 10.4049/jimmunol.0902388 19917686

[74] Stover CM Thiel S Thelen M Lynch NJ Vorup-Jensen T Jensenius JC Two constituents of the initiation complex of the mannan-binding lectin activation pathway of complement are encoded by a single structural gene. J Immunol (1999) 162(6):3481–90. 10092804

[75] Heja D Kocsis A Dobo J Szilagyi K Szasz R Zavodszky P Revised mechanism of complement lectin-pathway activation revealing the role of serine protease MASP-1 as the exclusive activator of MASP-2. Proc Natl Acad Sci U S A (2012) 109(26):10498–503. 10.1073/pnas.1202588109 PMC3387078 22691502

[76] Schwaeble W Dahl MR Thiel S Stover C Jensenius JC The mannan-binding lectin-associated serine proteases (MASPs) and MAp19: four components of the lectin pathway activation complex encoded by two genes. Immunobiology (2002) 205(4-5):455–66. 10.1078/0171-2985-00146 12396007

[77] Iwaki D Kanno K Takahashi M Endo Y Matsushita M Fujita T The role of mannose-binding lectin-associated serine protease-3 in activation of the alternative complement pathway. J Immunol (2011) 187(7):3751–8. 10.4049/jimmunol.1100280 21865552

[78] Fishelson Z Muller-Eberhard HJ Regulation of the alternative pathway of human complement by C1q. Mol Immunol (1987) 24(9):987–93. 10.1016/0161-5890(87)90011-3 2958692

[79] Fishelson Z Pangburn MK Muller-Eberhard HJ Characterization of the initial C3 convertase of the alternative pathway of human complement. J Immunol (1984) 132(3):1430–4. 6559201

[80] Fromell K Adler A Aman A Manivel VA Huang S Duhrkop C Assessment of the Role of C3(H2O) in the Alternative Pathway. Front Immunol (2020) 11:530. 10.3389/fimmu.2020.00530 32296436 PMC7136553

[81] Thurman JM Holers VM The central role of the alternative complement pathway in human disease. J Immunol (2006) 176(3):1305–10. 10.4049/jimmunol.176.3.1305 16424154

[82] Blatt AZ Pathan S Ferreira VP Properdin: a tightly regulated critical inflammatory modulator. Immunol Rev (2016) 274(1):172–90. 10.1111/imr.12466 PMC5096056 27782331

[83] Bayly-Jones C Bubeck D Dunstone MA The mystery behind membrane insertion: a review of the complement membrane attack complex. Philos Trans R Soc Lond B Biol Sci (2017) 372(1726):20160221. 10.1098/rstb.2016.0221 28630159 PMC5483522

[84] Schmidt CQ Lambris JD Ricklin D Protection of host cells by complement regulators. Immunol Rev (2016) 274(1):152–71. 10.1111/imr.12475 PMC5432642 27782321

[85] Skerka C Chen Q Fremeaux-Bacchi V Roumenina LT Complement factor H related proteins (CFHRs). Mol Immunol (2013) 56(3):170–80. 10.1016/j.molimm.2013.06.001 23830046

[86] Tschopp J French LE Clusterin: modulation of complement function. Clin Exp Immunol (1994) 97(Suppl 2):11–4. 10.1111/j.1365-2249.1994.tb06256.x PMC1550357 8070141

[87] Tschopp J Chonn A Hertig S French LE Clusterin, the human apolipoprotein and complement inhibitor, binds to complement C7, C8 beta, and the b domain of C9. J Immunol (1993) 151(4):2159–65. 8345200

[88] Davies A Simmons DL Hale G Harrison RA Tighe H Lachmann PJ CD59, an LY-6-like protein expressed in human lymphoid cells, regulates the action of the complement membrane attack complex on homologous cells. J Exp Med (1989) 170(3):637–54. 10.1084/jem.170.3.637 PMC2189447 2475570

[89] Ratnoff OD Lepow IH Some properties of an esterase derived from preparations of the first component of complement. J Exp Med (1957) 106(2):327–43. 10.1084/jem.106.2.327 PMC2136749 13449241

[90] Nilsson SC Sim RB Lea SM Fremeaux-Bacchi V Blom AM Complement factor I in health and disease. Mol Immunol (2011) 48(14):1611–20. 10.1016/j.molimm.2011.04.004 21529951

[91] Iida K Nussenzweig V Complement receptor is an inhibitor of the complement cascade. J Exp Med (1981) 153(5):1138–50. 10.1084/jem.153.5.1138 PMC2186151 6910481

[92] Nicholson-Weller A Wang CE Structure and function of decay accelerating factor CD55. J Lab Clin Med (1994) 123(4):485–91. 7511675

[93] Liszewski MK Post TW Atkinson JP Membrane cofactor protein (MCP or CD46): newest member of the regulators of complement activation gene cluster. Annu Rev Immunol (1991) 9:431–55. 10.1146/annurev.iy.09.040191.002243 1910685

[94] Parente R Clark SJ Inforzato A Day AJ Complement factor H in host defense and immune evasion. Cell Mol Life Sci (2017) 74(9):1605–24. 10.1007/s00018-016-2418-4 PMC5378756 27942748

[95] Wong EKS Kavanagh D Diseases of complement dysregulation-an overview. Semin Immunopathol (2018) 40(1):49–64. 10.1007/s00281-017-0663-8 29327071 PMC5794843

[96] Weed JC Arquembourg PC Endometriosis: can it produce an autoimmune response resulting in infertility? Clin Obstet Gynecol (1980) 23(3):885–93. 10.1097/00003081-198023030-00018 7418287

[97] Bartosik D Damjanov I Viscarello RR Riley JA Immunoproteins in the endometrium: clinical correlates of the presence of complement fractions C3 and C4. Am J Obstet Gynecol (1987) 156(1):11–5. 10.1016/0002-9378(87)90194-3 3541614

[98] Rekker K Saare M Eriste E Tasa T Kukuskina V Roost AM High-throughput mRNA sequencing of stromal cells from endometriomas and endometrium. Reproduction (2017) 154(1):93–100. 10.1530/REP-17-0092 28495852

[99] Suryawanshi S Huang X Elishaev E Budiu RA Zhang L Kim S Complement pathway is frequently altered in endometriosis and endometriosis-associated ovarian cancer. Clin Cancer Res (2014) 20(23):6163–74. 10.1158/1078-0432.CCR-14-1338 PMC4252715 25294912

[100] Signorile PG Baldi A Serum biomarker for diagnosis of endometriosis. J Cell Physiol (2014) 229(11):1731–5. 10.1002/jcp.24620 24648304

[101] Ruiz LA Dutil J Ruiz A Fourquet J Abac S Laboy J Single-nucleotide polymorphisms in the lysyl oxidase-like protein 4 and complement component 3 genes are associated with increased risk for endometriosis and endometriosis-associated infertility. Fertil Steril (2011) 96(2):512–5. 10.1016/j.fertnstert.2011.06.001 PMC3143268 21733505

[102] Bischof P Planas-Basset D Meisser A Campana A Investigations on the cell type responsible for the endometrial secretion of complement component 3 (C3). Hum Reprod (1994) 9(9):1652–9. 10.1093/oxfordjournals.humrep.a138768 7836514

[103] Isaacson KB Coutifaris C Garcia CR Lyttle CR Production and secretion of complement component 3 by endometriotic tissue. J Clin Endocrinol Metab (1989) 69(5):1003–9. 10.1210/jcem-69-5-1003 2793987

[104] Tao XJ Sayegh RA Isaacson KB Increased expression of complement component 3 in human ectopic endometrium compared with the matched eutopic endometrium. Fertil Steril (1997) 68(3):460–7. 10.1016/s0015-0282(97)00254-9 9314915

[105] Isaacson KB Xu Q Lyttle CR The effect of estradiol on the production and secretion of complement component 3 by the rat uterus and surgically induced endometriotic tissue. Fertil Steril (1991) 55(2):395–402. 10.1016/S0015-0282(16)54135-1 1991537

[106] D’Cruz OJ Wild RA Evaluation of endometrial tissue specific complement activation in women with endometriosis. Fertil Steril (1992) 57(4):787–95. 10.1016/S0015-0282(16)54960-7 1372870

[107] Markiewski MM Nilsson B Ekdahl KN Mollnes TE Lambris JD Complement and coagulation: strangers or partners in crime? Trends Immunol (2007) 28(4):184–92. 10.1016/j.it.2007.02.006 17336159

[108] Pawluczkowycz AW Lindorfer MA Waitumbi JN Taylor RP Hematin promotes complement alternative pathway-mediated deposition of C3 activation fragments on human erythrocytes: potential implications for the pathogenesis of anemia in malaria. J Immunol (2007) 179(8):5543–52. 10.4049/jimmunol.179.8.5543 17911641

[109] Harrison RA The properdin pathway: an “alternative activation pathway” or a “critical amplification loop” for C3 and C5 activation? Semin Immunopathol (2018) 40(1):15–35. 10.1007/s00281-017-0661-x 29167939

[110] Palomino WA Tayade C Argandona F Devoto L Young SL Lessey BA The endometria of women with endometriosis exhibit dysfunctional expression of complement regulatory proteins during the mid secretory phase. J Reprod Immunol (2018) 125:1–7. 10.1016/j.jri.2017.10.046 29153978

[111] Ahn SH Khalaj K Young SL Lessey BA Koti M Tayade C Immune-inflammation gene signatures in endometriosis patients. Fertil Steril (2016) 106(6):1420–31 e7. 10.1016/j.fertnstert.2016.07.005 27475412 PMC5683404

[112] Kobayashi H Yamashita Y Iwase A Yoshikawa Y Yasui H Kawai Y The ferroimmunomodulatory role of ectopic endometriotic stromal cells in ovarian endometriosis. Fertil Steril (2012) 98(2):415–22 e1-12. 10.1016/j.fertnstert.2012.04.047 22633261

[113] Fassbender A Burney RO Dorien FO D’Hooghe T Giudice L Update on Biomarkers for the Detection of Endometriosis. BioMed Res Int (2015) 2015:130854. 10.1155/2015/130854 26240814 PMC4512573

[114] Eisermann J Gast MJ Pineda J Odem RR Collins JL Tumor necrosis factor in peritoneal fluid of women undergoing laparoscopic surgery. Fertil Steril (1988) 50(4):573–9. 10.1016/s0015-0282(16)60185-1 2971579

[115] Bedaiwy MA Falcone T Peritoneal fluid environment in endometriosis. Clinicopathological implications. Minerva Ginecol (2003) 55(4):333–45. 14581858

[116] May KE Conduit-Hulbert SA Villar J Kirtley S Kennedy SH Becker CM Peripheral biomarkers of endometriosis: a systematic review. Hum Reprod Update (2010) 16(6):651–74. 10.1093/humupd/dmq009 PMC2953938 20462942

[117] Edwards RP Huang X Vlad AM Chronic inflammation in endometriosis and endometriosis-associated ovarian cancer: New roles for the “old” complement pathway. Oncoimmunology (2015) 4(5):e1002732. 10.1080/2162402X.2014.1002732 26155393 PMC4485759

[118] Agostinis C Zorzet S Balduit A Zito G Mangogna A Macor P Complement Component 3 expressed by the endometrial ectopic tissue is involved in the endometriotic lesion formation through mast cell activation. bioRxiv (2020), 2020.11.19.389536. 10.1101/2020.11.19.389536

[119] Kabut J Kondera-Anasz Z Sikora J Mielczarek-Palacz A Levels of complement components iC3b, C3c, C4, and SC5b-9 in peritoneal fluid and serum of infertile women with endometriosis. Fertil Steril (2007) 88(5):1298–303. 10.1016/j.fertnstert.2006.12.061 17482181

[120] Sikora J Wroblewska-Czech A Smycz-Kubanska M Mielczarek-Palacz A Cygal A Witek A The role of complement components C1q, MBL and C1 inhibitor in pathogenesis of endometriosis. Arch Gynecol Obstet (2018) 297(6):1495–501. 10.1007/s00404-018-4754-0 PMC5945730 29572748

[121] Fassbender A D’Hooghe T Mihalyi A Kyama C Simsa P Lessey BA Plasma C3a-des-Arg levels in women with and without endometriosis. Am J Reprod Immunol (2009) 62(3):187–95. 10.1111/j.1600-0897.2009.00728.x 19694644

[122] Zhang T De Carolis C Man GCW Wang CC The link between immunity, autoimmunity and endometriosis: a literature update. Autoimmun Rev (2018) 17(10):945–55. 10.1016/j.autrev.2018.03.017 30107265

[123] Csuka D Veszeli N Varga L Prohaszka Z Farkas H The role of the complement system in hereditary angioedema. Mol Immunol (2017) 89:59–68. 10.1016/j.molimm.2017.05.020 28595743

[124] Morgan BP Hereditary angioedema–therapies old and new. N Engl J Med (2010) 363(6):581–3. 10.1056/NEJMe1006450 20818894

[125] Rother RP Rollins SA Mojcik CF Brodsky RA Bell L Discovery and development of the complement inhibitor eculizumab for the treatment of paroxysmal nocturnal hemoglobinuria. Nat Biotechnol (2007) 25(11):1256–64. 10.1038/nbt1344 17989688

[126] Rosse WF Dacie JV Immune lysis of normal human and paroxysmal nocturnal hemoglobinuria (PNH) red blood cells. I. The sensitivity of PNH red cells to lysis by complement and specific antibody. J Clin Invest (1966) 45(5):736–48. 10.1172/JCI105388 PMC292750 5935361

[127] Rosse WF Dacie JV Immune lysis of normal human and paroxysmal nocturnal hemoglobinuria (PNH) red blood cells. II. The role of complement components in the increased sensitivity of PNH red cells to immune lysis. J Clin Invest (1966) 45(5):749–57. 10.1172/JCI105389 PMC292751 4956901

[128] Zelek WM Xie L Morgan BP Harris CL Compendium of current complement therapeutics. Mol Immunol (2019) 114:341–52. 10.1016/j.molimm.2019.07.030 31446305

[129] Brodsky RA Young NS Antonioli E Risitano AM Schrezenmeier H Schubert J Multicenter phase 3 study of the complement inhibitor eculizumab for the treatment of patients with paroxysmal nocturnal hemoglobinuria. Blood (2008) 111(4):1840–7. 10.1182/blood-2007-06-094136 18055865

[130] Harris CL Pouw RB Kavanagh D Sun R Ricklin D Developments in anti-complement therapy; from disease to clinical trial. Mol Immunol (2018) 102:89–119. 10.1016/j.molimm.2018.06.008 30121124

[131] Morgan BP Harris CL Complement, a target for therapy in inflammatory and degenerative diseases. Nat Rev Drug Discov (2015) 14(12):857–77. 10.1038/nrd4657 PMC7098197 26493766

[132] Ricklin D Mastellos DC Reis ES Lambris JD The renaissance of complement therapeutics. Nat Rev Nephrol (2018) 14(1):26–47. 10.1038/nrneph.2017.156 29199277 PMC5805379

[133] Sheridan D Yu ZX Zhang Y Patel R Sun F Lasaro MA Design and preclinical characterization of ALXN1210: A novel anti-C5 antibody with extended duration of action. PLoS One (2018) 13(4):e0195909. 10.1371/journal.pone.0195909 29649283 PMC5897016

[134] Igawa T Ishii S Tachibana T Maeda A Higuchi Y Shimaoka S Antibody recycling by engineered pH-dependent antigen binding improves the duration of antigen neutralization. Nat Biotechnol (2010) 28(11):1203–7. 10.1038/nbt.1691 20953198

[135] Fukuzawa T Sampei Z Haraya K Ruike Y Shida-Kawazoe M Shimizu Y Long lasting neutralization of C5 by SKY59, a novel recycling antibody, is a potential therapy for complement-mediated diseases. Sci Rep (2017) 7(1):1080. 10.1038/s41598-017-01087-7 28439081 PMC5430875

[136] Sampei Z Haraya K Tachibana T Fukuzawa T Shida-Kawazoe M Gan SW Antibody engineering to generate SKY59, a long-acting anti-C5 recycling antibody. PLoS One (2018) 13(12):e0209509. 10.1371/journal.pone.0209509 30592762 PMC6310256

[137] Risitano AM Marotta S Ricci P Marano L Frieri C Cacace F Anti-complement Treatment for Paroxysmal Nocturnal Hemoglobinuria: Time for Proximal Complement Inhibition? A Position Paper From the SAAWP of the EBMT. Front Immunol (2019) 10:1157. 10.3389/fimmu.2019.01157 31258525 PMC6587878

[138] Riedemann NC Habel M Ziereisen J Hermann M Schneider C Wehling C Controlling the anaphylatoxin C5a in diseases requires a specifically targeted inhibition. Clin Immunol (2017) 180:25–32. 10.1016/j.clim.2017.03.012 28366510

[139] Risitano AM Mastellos DC Huber-Lang M Yancopoulou D Garlanda C Ciceri F Complement as a target in COVID-19? Nat Rev Immunol (2020) 20(6):343–4. 10.1038/s41577-020-0320-7 PMC7187144 32327719

[140] Mastaglio S Ruggeri A Risitano AM Angelillo P Yancopoulou D Mastellos DC The first case of COVID-19 treated with the complement C3 inhibitor AMY-101. Clin Immunol (2020) 215:108450. 10.1016/j.clim.2020.108450 32360516 PMC7189192

[141] Rambaldi A Gritti G Micò MC Frigeni M Borleri G Salvi A Endothelial Injury and Thrombotic Microangiopathy in COVID-19: Treatment with the Lectin-Pathway Inhibitor Narsoplimab. Immunobiology (2020) 225(6):152001. 10.1016/j.imbio.2020.152001 32943233 PMC7415163

[142] Mastellos DC Reis ES Ricklin D Smith RJ Lambris JD Complement C3-Targeted Therapy: Replacing Long-Held Assertions with Evidence-Based Discovery. Trends Immunol (2017) 38(6):383–94. 10.1016/j.it.2017.03.003 PMC5447467 28416449

[143] Wong RS Pullon HWH Deschatelets P Francois CG Hamdani M Issaragrisil S Inhibition of C3 with APL-2 Results in Normalisation of Markers of Intravascular and Extravascular Hemolysis in Patients with Paroxysmal Nocturnal Hemoglobinuria (PNH). Blood (2018) 132(Supplement 1):2314–. 10.1182/blood-2018-99-110827

[144] Grossi F Shum MK Gertz MA Roman E Deschatelets P Hamdani M Inhibition of C3 with APL-2 Results in Normalisation of Markers of Intravascular and Extravascular Hemolysis in Patients with Autoimmune Hemolytic Anemia (AIHA). Blood (2018) 132(Supplement 1):3623–. 10.1182/blood-2018-99-119468

[145] Grossi FV Bedwell P Deschatelets P Edis L Francois CG Johnson PJ APL-2, a Complement C3 Inhibitor for the Potential Treatment of Paroxysmal Nocturnal Hemoglobinuria (PNH): Phase I Data from Two Completed Studies in Healthy Volunteers. Blood (2016) 128(22):1251–. 10.1182/blood.V128.22.1251.1251

[146] Chow V Pan J Chien D Mytych DT Hanes V A randomized, double-blind, single-dose, three-arm, parallel group study to determine pharmacokinetic similarity of ABP 959 and eculizumab (Soliris((R)) ) in healthy male subjects. Eur J Haematol (2020) 105(1):66–74. 10.1111/ejh.13411 32196749 PMC7384155

[147] Merkel PA Jayne DR Wang C Hillson J Bekker P Evaluation of the Safety and Efficacy of Avacopan, a C5a Receptor Inhibitor, in Patients With Antineutrophil Cytoplasmic Antibody-Associated Vasculitis Treated Concomitantly With Rituximab or Cyclophosphamide/Azathioprine: Protocol for a Randomized, Double-Blind, Active-Controlled, Phase 3 Trial. JMIR Res Protoc (2020) 9(4):e16664. 10.2196/16664 32088663 PMC7175182

[148] Tesar V Hruskova Z Avacopan in the treatment of ANCA-associated vasculitis. Expert Opin Invest Drugs (2018) 27(5):491–6. 10.1080/13543784.2018.1472234 29718732

[149] Jayne DRW Bruchfeld AN Harper L Schaier M Venning MC Hamilton P Randomized Trial of C5a Receptor Inhibitor Avacopan in ANCA-Associated Vasculitis. J Am Soc Nephrol (2017) 28(9):2756–67. 10.1681/ASN.2016111179 PMC5576933 28400446

[150] Bekker P Dairaghi D Seitz L Leleti M Wang Y Ertl L Characterization of Pharmacologic and Pharmacokinetic Properties of CCX168, a Potent and Selective Orally Administered Complement 5a Receptor Inhibitor, Based on Preclinical Evaluation and Randomized Phase 1 Clinical Study. PLoS One (2016) 11(10):e0164646. 10.1371/journal.pone.0164646 27768695 PMC5074546

[151] Keating GM Human C1-esterase inhibitor concentrate (Berinert). BioDrugs (2009) 23(6):399–406. 10.2165/11201100-000000000-00000 19894781

[152] Zanichelli A Azin GM Cristina F Vacchini R Caballero T Safety, effectiveness, and impact on quality of life of self-administration with plasma-derived nanofiltered C1 inhibitor (Berinert(R)) in patients with hereditary angioedema: the SABHA study. Orphanet J Rare Dis (2018) 13(1):51. 10.1186/s13023-018-0797-3 29631595 PMC5891972

[153] Aygoren-Pursun E Soteres D Moldovan D Christensen J Van Leerberghe A Hao J Preventing Hereditary Angioedema Attacks in Children Using Cinryze(R): Interim Efficacy and Safety Phase 3 Findings. Int Arch Allergy Immunol (2017) 173(2):114–9. 10.1159/000477541 PMC5841133 28662509

[154] Lyseng-Williamson KA Nanofiltered human C1 inhibitor concentrate (Cinryze(R)): in hereditary angioedema. BioDrugs (2011) 25(5):317–27. 10.2165/11208390-000000000-00000 21942916

[155] Bernstein JA Manning ME Li H White MV Baker J Lumry WR Escalating doses of C1 esterase inhibitor (CINRYZE) for prophylaxis in patients with hereditary angioedema. J Allergy Clin Immunol Pract (2014) 2(1):77–84. 10.1016/j.jaip.2013.09.008 24565773

[156] Roth A Nishimura JI Nagy Z Gaal-Weisinger J Panse J Yoon SS The complement C5 inhibitor crovalimab in paroxysmal nocturnal hemoglobinuria. Blood (2020) 135(12):912–20. 10.1182/blood.2019003399 PMC7082616 31978221

[157] Wiles JA Galvan MD Podos SD Geffner M Huang M Discovery and Development of the Oral Complement Factor D Inhibitor Danicopan (ACH-4471). Curr Med Chem (2020) 27(25):4165–80. 10.2174/0929867326666191001130342 31573880

[158] Risitano AM Kulasekararaj AG Lee JW Maciejewski JP Notaro R Brodsky R Danicopan: an oral complement factor D inhibitor for paroxysmal nocturnal hemoglobinuria. Haematologica (2020). 10.3324/haematol.2020.261826 PMC8634185 33121236

[159] Wijnsma KL Ter Heine R Moes D Langemeijer S Schols SEM Volokhina EB Pharmacology, Pharmacokinetics and Pharmacodynamics of Eculizumab, and Possibilities for an Individualized Approach to Eculizumab. Clin Pharmacokinet (2019) 58(7):859–74. 10.1007/s40262-019-00742-8 PMC6584251 30758736

[160] Giamarellos-Bourboulis EJ Argyropoulou M Kanni T Spyridopoulos T Otto I Zenker O Clinical efficacy of complement C5a inhibition by IFX-1 in hidradenitis suppurativa: an open-label single-arm trial in patients not eligible for adalimumab. Br J Dermatol (2020) 183(1):176–8. 10.1111/bjd.18877 31954061

[161] Vlaar APJ de Bruin S Busch M Timmermans S van Zeggeren IE Koning R Anti-C5a antibody IFX-1 (vilobelimab) treatment versus best supportive care for patients with severe COVID-19 (PANAMO): an exploratory, open-label, phase 2 randomised controlled trial. Lancet Rheumatol (2020) 2(12):e764–73. 10.1016/S2665-9913(20)30341-6 PMC7521913 33015643

[162] Halstead SK Humphreys PD Goodfellow JA Wagner ER Smith RA Willison HJ Complement inhibition abrogates nerve terminal injury in Miller Fisher syndrome. Ann Neurol (2005) 58(2):203–10. 10.1002/ana.20546 16049921

[163] Kassimatis T Qasem A Douiri A Ryan EG Rebollo-Mesa I Nichols LL A double-blind randomised controlled investigation into the efficacy of Mirococept (APT070) for preventing ischaemia reperfusion injury in the kidney allograft (EMPIRIKAL): study protocol for a randomised controlled trial. Trials (2017) 18(1):255. 10.1186/s13063-017-1972-x 28587616 PMC5461672

[164] Schols S Nunn MA Mackie I Weston-Davies W Nishimura JI Kanakura Y Successful treatment of a PNH patient non-responsive to eculizumab with the novel complement C5 inhibitor coversin (nomacopan). Br J Haematol (2020) 188(2):334–7. 10.1111/bjh.16305 31840801

[165] Elhadad S Chapin J Copertino D Van Besien K Ahamed J Laurence J MASP2 levels are elevated in thrombotic microangiopathies: association with microvascular endothelial cell injury and suppression by anti-MASP2 antibody narsoplimab. Clin Exp Immunol (2020). 10.1111/cei.13497 PMC7405159 32681658

[166] Selvaskandan H Kay Cheung C Dormer J Wimbury D Martinez M Xu G Inhibition of the Lectin Pathway of the Complement System as a Novel Approach in the Management of IgA Vasculitis-Associated Nephritis. Nephron (2020) 144(9):453–8. 10.1159/000508841 32721954

[167] Latuszek A Liu Y Olsen O Foster R Cao M Lovric I Inhibition of complement pathway activation with Pozelimab, a fully human antibody to complement component C5. PLoS One (2020) 15(5):e0231892. 10.1371/journal.pone.0231892 32384086 PMC7209288

[168] Busse PJ Christiansen SC Hereditary Angioedema. N Engl J Med (2020) 382(12):1136–48. 10.1056/NEJMra1808012 32187470

[169] Cruz MP Conestat alfa (ruconest): first recombinant c1 esterase inhibitor for the treatment of acute attacks in patients with hereditary angioedema. P T (2015) 40(2):109–14. PMC4315111 25673959

[170] Freire PC Munoz CH Derhaschnig U Schoergenhofer C Firbas C Parry GC Specific Inhibition of the Classical Complement Pathway Prevents C3 Deposition along the Dermal-Epidermal Junction in Bullous Pemphigoid. J Invest Dermatol (2019) 139(12):2417–24 e2. 10.1016/j.jid.2019.04.025 31229501

[171] Nikitin PA Rose EL Byun TS Parry GC Panicker S C1s Inhibition by BIVV009 (Sutimlimab) Prevents Complement-Enhanced Activation of Autoimmune Human B Cells In Vitro. J Immunol (2019) 202(4):1200–9. 10.4049/jimmunol.1800998 PMC6360260 30635392

[172] Bartko J Schoergenhofer C Schwameis M Firbas C Beliveau M Chang C A Randomized, First-in-Human, Healthy Volunteer Trial of sutimlimab, a Humanized Antibody for the Specific Inhibition of the Classical Complement Pathway. Clin Pharmacol Ther (2018) 104(4):655–63. 10.1002/cpt.1111 PMC6175298 29737533

[173] Jager U D’Sa S Schorgenhofer C Bartko J Derhaschnig U Sillaber C Inhibition of complement C1s improves severe hemolytic anemia in cold agglutinin disease: a first-in-human trial. Blood (2019) 133(9):893–901. 10.1182/blood-2018-06-856930 30559259 PMC6396179

[174] Jordan SC Kucher K Bagger M Hockey HU Wagner K Ammerman N Intravenous immunoglobulin significantly reduces exposure of concomitantly administered anti-C5 monoclonal antibody tesidolumab. Am J Transplant (2020) 20(9):2581–8. 10.1111/ajt.15922 32301258

[175] Albazli K Kaminski HJ Howard JF Jr. Complement Inhibitor Therapy for Myasthenia Gravis. Front Immunol (2020) 11:917. 10.3389/fimmu.2020.00917 32582144 PMC7283905

[176] Wilkinson T Dixon R Page C Carroll M Griffiths G Ho LP ACCORD: A Multicentre, Seamless, Phase 2 Adaptive Randomisation Platform Study to Assess the Efficacy and Safety of Multiple Candidate Agents for the Treatment of COVID-19 in Hospitalised Patients: A structured summary of a study protocol for a randomised controlled trial. Trials (2020) 21(1):691. 10.1186/s13063-020-04584-9 32736596 PMC7393340

[177] Oosterlynck DJ Meuleman C Waer M Vandeputte M Koninckx PR The natural killer activity of peritoneal fluid lymphocytes is decreased in women with endometriosis. Fertil Steril (1992) 58(2):290–5. 10.1016/s0015-0282(16)55224-8 1633893

[178] Oosterlynck DJ Cornillie FJ Waer M Vandeputte M Koninckx PR Women with endometriosis show a defect in natural killer activity resulting in a decreased cytotoxicity to autologous endometrium. Fertil Steril (1991) 56(1):45–51. 10.1016/s0015-0282(16)54414-8 2065804

[179] Thiruchelvam U Wingfield M O’Farrelly C Natural Killer Cells: Key Players in Endometriosis. Am J Reprod Immunol (2015) 74(4):291–301. 10.1111/aji.12408 26104509

[180] Jeung I Cheon K Kim MR Decreased Cytotoxicity of Peripheral and Peritoneal Natural Killer Cell in Endometriosis. BioMed Res Int (2016) 2016:2916070. 10.1155/2016/2916070 27294113 PMC4880704

[181] Sciezynska A Komorowski M Soszynska M Malejczyk J NK Cells as Potential Targets for Immunotherapy in Endometriosis. J Clin Med (2019) 8(9):1468. 10.3390/jcm8091468 PMC6780982 31540116

[182] Chen Z Yang Y Liu LL Lundqvist A Strategies to Augment Natural Killer (NK) Cell Activity against Solid Tumors. Cancers (Basel) (2019) 11(7):1040. 10.3390/cancers11071040 PMC6678934 31340613

[183] Sivori S Vacca P Del Zotto G Munari E Mingari MC Moretta L Human NK cells: surface receptors, inhibitory checkpoints, and translational applications. Cell Mol Immunol (2019) 16(5):430–41. 10.1038/s41423-019-0206-4 PMC6474200 30778167

[184] Liu CF Min XY Wang N Wang JX Ma N Dong X Complement Receptor 3 Has Negative Impact on Tumor Surveillance through Suppression of Natural Killer Cell Function. Front Immunol (2017) 8:1602. 10.3389/fimmu.2017.01602 29209332 PMC5702005

[185] Vivier E Tomasello E Baratin M Walzer T Ugolini S Functions of natural killer cells. Nat Immunol (2008) 9(5):503–10. 10.1038/ni1582 18425107

[186] Son M Porat A He M Suurmond J Santiago-Schwarz F Andersson U C1q and HMGB1 reciprocally regulate human macrophage polarization. Blood (2016) 128(18):2218–28. 10.1182/blood-2016-05-719757 PMC5095756 27683415

[187] Spivia W Magno PS Le P Fraser DA Complement protein C1q promotes macrophage anti-inflammatory M2-like polarization during the clearance of atherogenic lipoproteins. Inflammation Res (2014) 63(10):885–93. 10.1007/s00011-014-0762-0 PMC5154336 25091012

[188] Bossi F Tripodo C Rizzi L Bulla R Agostinis C Guarnotta C C1q as a unique player in angiogenesis with therapeutic implication in wound healing. Proc Natl Acad Sci USA (2014) 111(11):4209–14. 10.1073/pnas.1311968111 PMC3964125 24591625

[189] Bulla R Tripodo C Rami D Ling GS Agostinis C Guarnotta C C1q acts in the tumour microenvironment as a cancer-promoting factor independently of complement activation. Nat Commun (2016) 7:10346. 10.1038/ncomms10346 26831747 PMC4740357

[190] Mangogna A Agostinis C Bonazza D Belmonte B Zacchi P Zito G Is the Complement Protein C1q a Pro- or Anti-tumorigenic Factor? Bioinformatics Analysis Involving Human Carcinomas. Front Immunol (2019) 10:865. 10.3389/fimmu.2019.00865 31130944 PMC6509152

